# Heterotypic CAF-tumor spheroids promote early peritoneal metastatis of ovarian cancer

**DOI:** 10.1084/jem.20180765

**Published:** 2019-03-04

**Authors:** Qinglei Gao, Zongyuan Yang, Sen Xu, Xiaoting Li, Xin Yang, Ping Jin, Yi Liu, Xiaoshui Zhou, Taoran Zhang, Cheng Gong, Xiao Wei, Dan Liu, Chaoyang Sun, Gang Chen, Junbo Hu, Li Meng, Jianfeng Zhou, Kenjiro Sawada, Robert Fruscio, Thomas W. Grunt, Jörg Wischhusen, Víctor Manuel Vargas-Hernández, Bhavana Pothuri, Robert L. Coleman

**Affiliations:** 1 Cancer Biology Research Center (Key Laboratory of the Ministry of Education), Tongji Hospital, Tongji Medical College, Huazhong University of Science and Technology, Wuhan, Hubei, China; 2 Department of Hematology, Tongji Hospital, Tongji Medical College, Huazhong University of Science and Technology, Wuhan, Hubei, China; 3 Department of Obstetrics and Gynecology, Osaka University Graduate School of Medicine, Yamadaoka Suita, Osaka, Japan; 4 Clinic of Obstetrics and Gynecology, San Gerardo Hospital, Monza, Italy; 5 Department of Medicine and Surgery, University of Milan-Bicocca, Milan, Italy; 6 Signaling Networks Program, Division of Oncology, Department of Medicine I, Comprehensive Cancer Center & Ludwig Boltzmann Cluster Oncology, Medical University of Vienna, Vienna, Austria; 7 Department of Obstetrics and Gynecology, Experimental Tumor Immunology, University of Würzburg Medical School, Würzburg, Germany; 8 Dirección de Investigación, Hospital Juárez de México D.F., Nápoles, Mexico City, Mexico; 9 Division of Gynecological Oncology, NYU Langone Medical Center, Perlmutter Cancer Center, New York, NY; 10 Department of Gynecological Oncology & Reproductive Medicine, University of Texas, M.D. Anderson Cancer Center, Houston, TX

## Abstract

High-grade serous ovarian cancer (HGSOC) is hallmarked by early onset of peritoneal dissemination, which distinguishes it from low-grade serous ovarian cancer (LGSOC). Here, we describe the aggressive nature of HGSOC ascitic tumor cells (ATCs) characterized by integrin α5^high^ (ITGA5^high^) ATCs, which are prone to forming heterotypic spheroids with fibroblasts. We term these aggregates as metastatic units (MUs) in HGSOC for their advantageous metastatic capacity and active involvement in early peritoneal dissemination. Intriguingly, fibroblasts inside MUs support ATC survival and guide their peritoneal invasion before becoming essential components of the tumor stroma in newly formed metastases. Cancer-associated fibroblasts (CAFs) recruit ITGA5^high^ ATCs to form MUs, which further sustain ATC ITGA5 expression by EGF secretion. Notably, LGSOC is largely devoid of CAFs and the resultant MUs, which might explain its metastatic delay. These findings identify a specialized MU architecture that amplifies the tumor–stroma interaction and promotes transcoelomic metastasis in HGSOC, providing the basis for stromal fibroblast-oriented interventions in hampering OC peritoneal propagation.

## Abstract (precis)

The study provides insights in HGSOC by identifying that ascitic CAFs selectively recruit ITGA5^high^ ascitic tumor cells to form heterotypic spheroids named metastatic units (MUs), which actively engage in peritoneal metastasis, discriminates HGSOC from LGSOC, and act as therapeutic targets in hampering OC metastasis.

## Graphical Abstract

**Figure:**
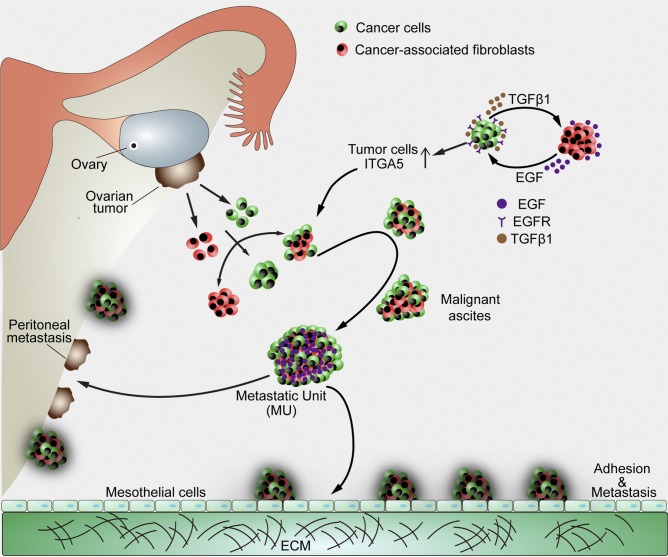

## Introduction

High-grade serous ovarian cancer (HGSOC)—the most aggressive form of ovarian cancer (OC)—is characterized by insidious onset, rapid i.p. spread, and the development of massive ascites (Vaughan et al., 2011; Konecny et al., 2014; Nik et al., 2014). However, its low-grade serous ovarian cancer (LGSOC) counterpart progresses slowly and has a more favorable outcome (Schmeler and Gershenson, 2008; Imamura et al., 2015). Although their distinct molecular origins have been elucidated, the mechanisms mediating this discrepant biology relative to peritoneal spreading are poorly understood (Tung et al., 2009; Angarita et al., 2015). In any case, to establish peritoneal metastases, tumor cells must escape from the primary tumor site as either single cells or spheroids (Wintzell et al., 2012; Auer et al., 2017), adhere to the mesothelial layer covering the abdominal cavity, and subsequently invade the favored extracellular matrix (ECM)–rich compartment (Kenny et al., 2011, 2015). Previous studies had emphasized that ascitic spheroids represent the invasive and chemoresistant cellular population fundamental to metastatic dissemination (Barbone et al., 2008; Sodek et al., 2009; Lawrenson et al., 2011). Little attention, however, has been devoted to analyzing tumor spheroid composition and ascitic tumor cell (ATC) heterogeneity in HGSOC patients and even less so in LGSOC patients. Considering the crucial role of peritoneal adhesion and the proposed function of spheroids during OC metastasis, we sought to investigate the processes by which ATCs assemble to form ascitic spheroids and subsequently execute peritoneal dissemination.

Ascites represents a tumor microenvironment that is rich in various cellular elements, cytokines, and ECM components (Ahmed and Stenvers, 2013; Thibault et al., 2014; Chudecka-Głaz et al., 2015). Development of ascites typically occurs upon dissemination of tumor cells into the peritoneum, before implantation of solid metastases. Ascites development is associated with disease progression in HGSOC patients (Kipps et al., 2013). Constant interactions between tumor cells and other components within the ascites fluid could significantly shape the malignant phenotype. For instance, ascites sustains a high percentage of cancer stem cells that contribute to disease recurrence and chemoresistance (Bapat et al., 2005; Latifi et al., 2012). Biomechanical factors such as fluidic force perturbations can also contribute to further tumor cell dissemination and metastatic progression (Rizvi et al., 2013). Importantly, the harsh hypoxic and anoikis-prone ascitic environment exerts a strong selective pressure on ATCs, thus allowing only the fittest cells to survive. Recently, tumor-associated macrophages (TAMs) were shown to drive spheroid formation and transcoelomic metastasis (Yin et al., 2016). Such mechanistic insights indicate that the ascites microenvironment can serve as a valuable platform to characterize ATCs with the intention of developing more effective therapies.

Therefore, we set out to investigate the intrinsic heterogeneity of ATCs and their contribution to OC progression. Our present study for the first time describes a critical role of cancer-associated fibroblast (CAF)–centered heterotypic spheroids, which represent metastatic units (MUs) in OC peritoneal adhesion and metastasis. The stromal fibroblast backbone recruits detached ATCs to form MUs at early stages of transcoelomic metastasis. We uncovered that integrin α5 (ITGA5) is indispensable for ATCs in forming MUs with CAFs. Moreover, epidermal growth factor (EGF) derived from activated fibroblasts inside the compact MU microenvironment further sustains ATC ITGA5 expression, which strengthens tumor–stromal interaction inside MUs. Our results thus imply that different interactions with ascitic CAFs and the resultant MU architecture might underlie the distinct patterns of peritoneal metastasis in HGSOC and LGSOC.

## Results

### HGSOC ATCs display an aggressive nature

**Figure 1.**
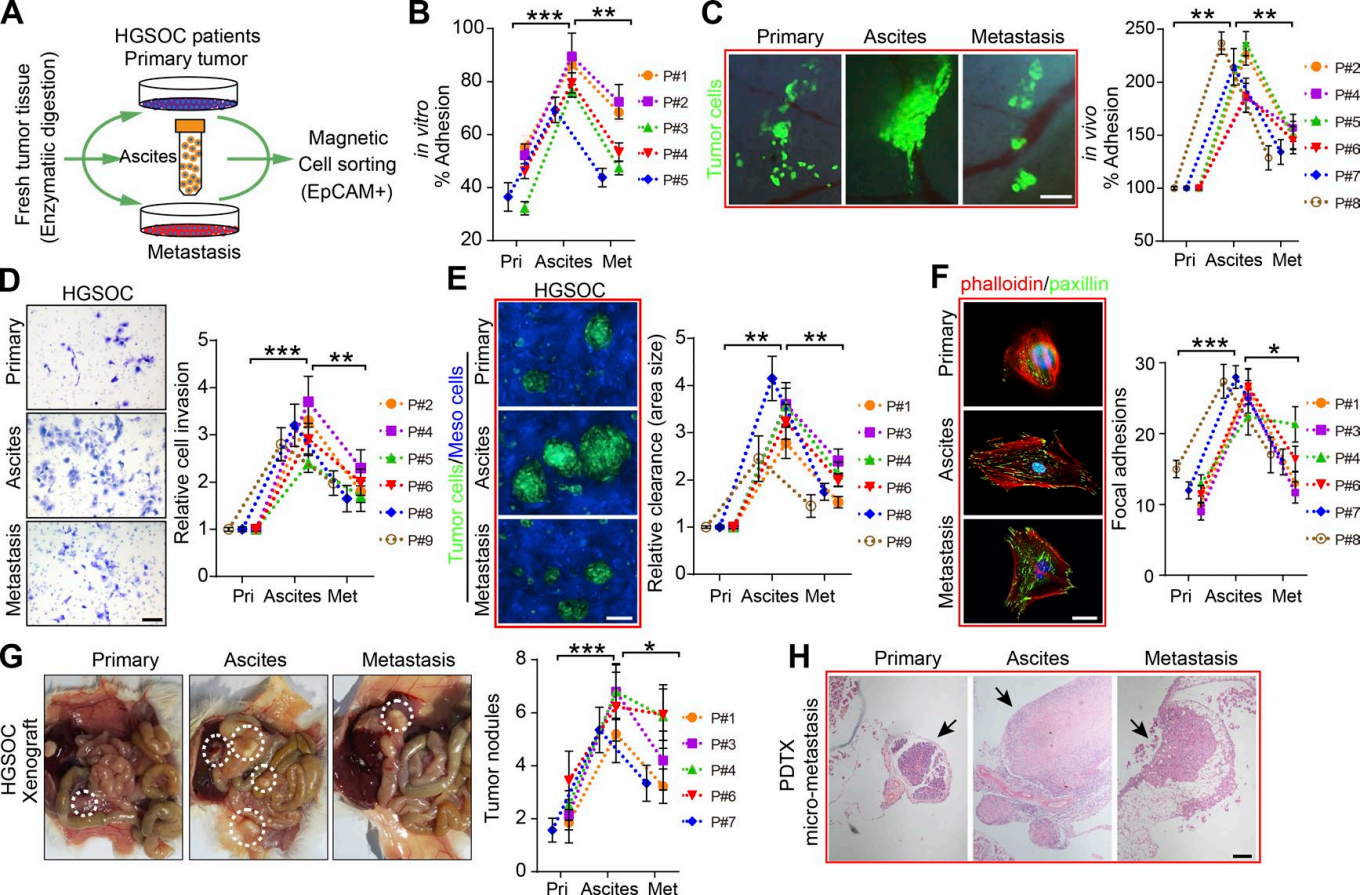
**ATCs are more adhesive and invasive than their matched counterparts in HGSOC patients. (A)** Scheme depicting the EpCAM microbead-based magnetic sorting of epithelial tumor cells from primary tumors, ascites, and metastases from HGSOC patients. **(B)** Relative percentage of paired tumor cells from primary tumors (Pri), ascites, and metastases (Met) adhesion to the major ECM protein fibronectin in HGSOC patients (#1–5). **(C–E)** Representative images and quantification of paired tumor cell adhesion (#2, 4, and 5–8) in murine abdominal cavity (C), tumor cell invasion (#2, 4–6, 8, and 9; D), and mesothelial clearance in HGSOC patients (#1, 3, 4, 6, 8, and 9; E). Bars, 200 µm (C), 50 µm (D), and 100 µm (E). **(F)** Immunofluorescence of the cytoskeleton (phalloidin) and FAs (paxillin) in paired tumor cells (#1, 3, 4, and 6–8) after exposure to a fibronectin-coated substrate FA measurements; *n* = 20. Bar, 20 µm. **(G)** Representative images and quantification of patient-derived tumor xenograft formation in mice implanted intraperitoneally with an equivalent number of paired tumor cells (#1, 3, 4, 6, and 7; *n* = 6 mice per group). **(H)** Representative H&E staining images of micrometastases formed by intraperitoneal implantation of equal amounts of paired tumor cells (#7). Bar, 200 µm. Data are means ± SEM and representative of four (B–F) or two (G and H) independent experiments. *, P < 0.05; **, P < 0.01; ***, P < 0.001; determined by paired Student’s *t* test.

To investigate the metastatic potential of ascites-derived tumor cells (ATCs), we isolated tumor epithelial cells from matched primary tumors, ascites, and solid metastases of HGSOC patients (Fig. 1 A). In vitro and in vivo adhesion assays both revealed that ATCs adhered more rapidly and securely to ECM substrate than matched primary and metastatic tumor cells (Fig. 1, B and C). Further analysis showed that ATCs were more invasive and exhibited enhanced mesothelial clearance capacity (Fig. 1, D and E), the latter of which is necessary for metastatic tumor growth (Aslan et al., 2015; Huang et al., 2015).

The development of tumor nodules from these penetrated malignant seeds requires focal adhesion (FA) and subsequent mesenchymal–epithelial transition that determines the fate of the attached tumor cells (Kenny et al., 2009; Lengyel, 2010). Correspondingly, we found that ATCs expressed more paxillin and developed robust FAs (Fig. 1 F). These cells also formed larger patient-derived xenograft tumors and more micrometastases compared with their counterparts from primary tumor and metastases (Fig. 1, G and H). In the context of LGSOC—an OC subtype that is clinically and molecularly distinct from HGSOC (Diaz-Padilla et al., 2012)—parallel experiments were performed. These revealed that ATCs displayed nearly equal capacity with respect to adhesion, invasion, and mesothelial clearance, compared with their matched counterparts (Fig. S1, A–D).

### ITGA5^high^ ATCs are fundamental to OC peritoneal metastasis

**Figure 2.**
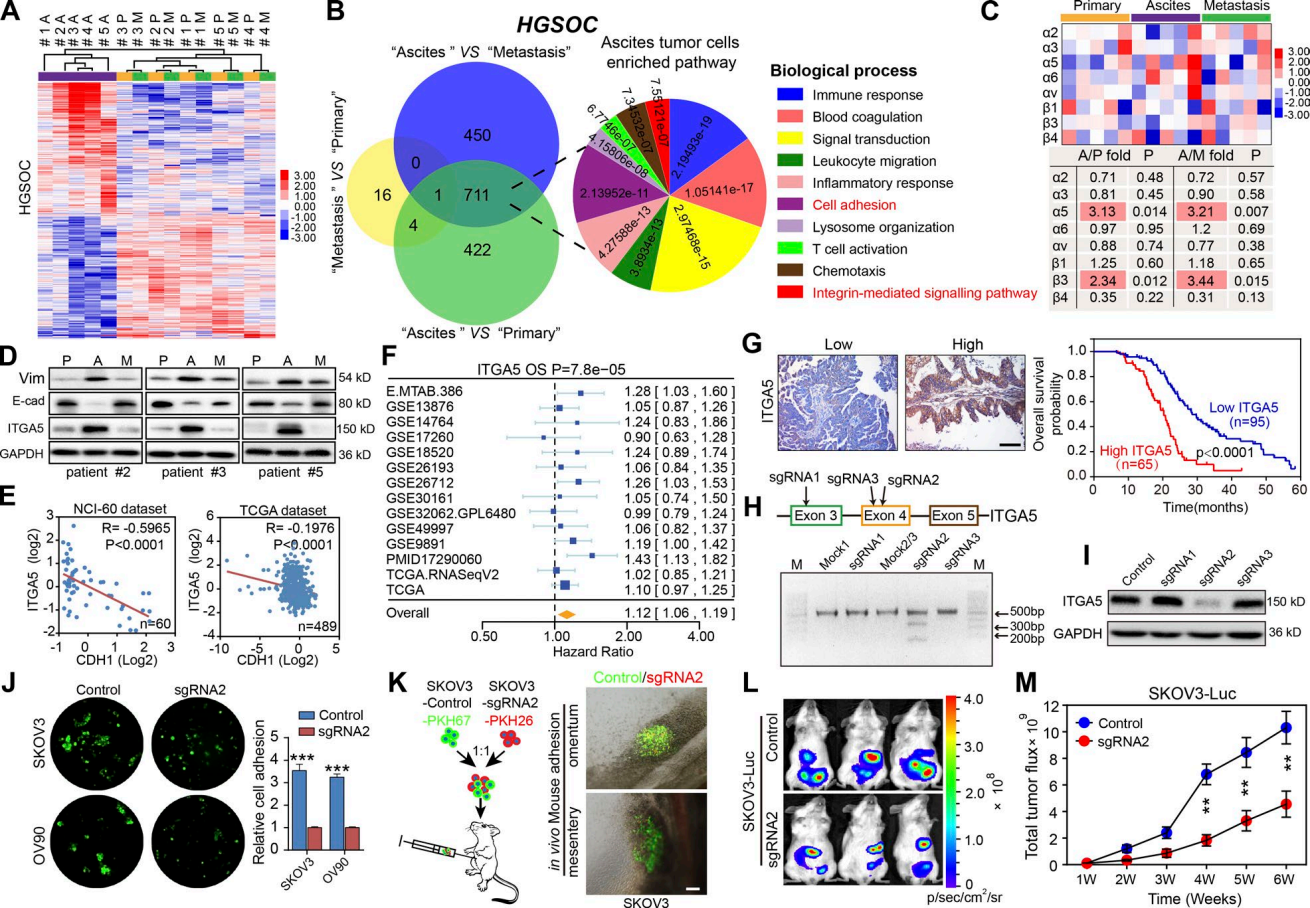
**ITGA5^high^ ATCs are crucial to OC peritoneal metastasis. (A)** Hierarchical clustering of differentially expressed genes among 15 epithelial tumor cell specimens isolated from the primary tumors (P), ascites (A), and metastases (M) of HGSOC patients (#1–5). **(B)** Venn diagram of the common signature genes differentially expressed between tumor cells of different origins and major biological processes represented in the gene sets specifically elevated in ATCs with their related statistical significances. **(C)** Expression levels of the relevant integrin family members in tumor cells of different origins. **(D)** Immunoblotting of ITGA5, E-cadherin, and Vim in freshly isolated paired tumor cells. **(E)** Pearson correlation analysis between ITGA5 and CDH1 mRNA expression in NCI-60 cells and in OC patient tissues from the TCGA dataset. **(F)** Meta-analysis depicting forest plots of ITGA5 expression as a univariate predictor of overall survival (OS). **(G)** Left: Representative images of low and high ITGA5 expression in OC tumor tissues. Right: Kaplan–Meier curves for overall survival probability in 160 serous OC patients with low (*n* = 95) and high (*n* = 65) ITGA5 protein expression (analyzed with log-rank test). Bar, 100 µm. **(H)** Editing ITGA5 in SKOV3 using CRISPR/Cas9 system. Top: Schematic diagram of sgRNAs 1–3 targeting exon 3/4 of ITGA5. Bottom: The genomic PCR products of SKOV3 cells transduced with scrambled sgRNA (mock) or sgRNA1–3 were analyzed with a T7E1 assay. **(I)** SKOV3 cells transduced with scrambled sgRNA (control) or sgRNA1-3 were subjected to immunoblotting with ITGA5. **(J)** Analyses of in vitro adhesion of SKOV3 or OV90 cells in the control or sgRNA2 group. **(K)** Schematic illustration of in vivo adhesion for equivalent numbers of PKH-67–labeled control and PKH-26–labeled sgRNA2 transduced SKOV3. Bar, 100 µm. **(L and M)** Representative bioluminescence images (L) and growth curves (M) developed by control SKOV3 and ITGA5-deficient (sgRNA2 group) SKOV3 cells (*n* = 10 mice per group). Data are means ± SEM. Data are one experiment (A–C and E–G) and representative of two (D, H, I, L, and M) or three (J and K) independent experiments. **, P < 0.01; ***, P < 0.001, determined by Student’s *t* test.

The distinct characteristics of ATCs drove us to further explore their specific phenotype. After isolation of matched tumor epithelial cells from primary, ascites, and omentum metastatic loci from HGSOC patients, the transcriptome of ATCs clearly differed from primary and metastatic tumor cells, which were quite similar to each other (Fig. 2 A). The comparison between ATCs and either primary or metastatic tumor cells revealed 1,138 and 1,162 genes, respectively, to be overexpressed twofold or more in ATCs (Tables S1 and S2), whereas only 21 genes were upregulated twofold or more between metastatic and primary tumor cells (Table S3). The overlapping set of 712 genes overexpressed in the ATCs was enriched for genes involved in the immune response, cell adhesion, and integrin-mediated signaling (Fig. 2 B). A similar comparison with cells derived from patients harboring LGSOC revealed that cells in the primary tumor showed a gene expression pattern which was distinct from that of ascites and metastatic tumor cells, whereas the transcriptional profile of ATCs was highly comparable to that of metastatic tumor cells. The most pronounced genetic variation in LGSOC was observed between metastatic and primary tumor cells, in which the up-regulated gene sets were enriched for genes involved in cell cycle regulation, cell adhesion, and multicellular organismal development. No significant functional enrichment was noted in the genes elevated in ATCs compared with either primary or metastatic tumor cells (Fig. S1, E and F). As previous studies highlighted the pivotal role of the integrin family in mediating OC peritoneal adhesion and metastasis (Casey and Skubitz, 2000; Elmasri et al., 2009), we further analyzed the expression of the over-represented integrins and observed that ITGα5 and β3 were specifically up-regulated in HGSOC ATCs (Fig. 2 C), whereas no specific dominant integrin expression was noted in LGSOC ATCs (Fig. S1 G). ITGA5 was selected for further analysis due to its recognized role in adhesion regulation and correlation with worse patient outcome in OC (Sawada et al., 2008; Mitra et al., 2011).

Enhanced expression of ITGA5 and vimentin (Vim) and reduced CDH1 (E-cadherin) expression were confirmed by immunoblotting in matched tumor epithelial cells (Fig. 2 D). The inverse correlation between ITGA5 and the epithelial marker CDH1 was further validated in NCI-60 cells (*R* = −0.5965, P < 0.0001) and The Cancer Genome Atlas (TCGA) datasets (*R* = –0.1976, P < 0.0001; Fig. 2 E), indicating that ITGA5^high^ cells represent a mesenchymal OC subpopulation. Both ITGA5 mRNA and protein levels correlated with worse patient outcome in public databases and in our panel of HGSOC specimens (Fig. 2, F and G). To assess the effects of loss of ITGA5 on tumor growth in vivo, we used CRISPR/Cas9 technology to knock down ITGA5 in SKOV3 cells. Three single guide RNAs (sgRNAs) were designed, and ablation of ITGA5 was most evident in tumor cells edited with sgRNA2, confirmed with T7 endonuclease I (T7E1) assay and immunoblotting (Fig. 2, H and I). Subsequently, ITGA5-deficient cells (sgRNA2 group) were observed to form smaller aggregates and displayed a markedly decreased capacity for adhesion compared with control HGSOC cell line OV90 and the ascites-derived ovarian adenocarcinoma cell line SKOV3 (Fig. 2 J). In addition, an in vivo assay further revealed that the majority of cells adhering to the metastatic “tropism” omentum and mesentery were PKH-67–labeled ITGA5 complete cells (Fig. 2 K). Finally, bioluminescence imaging revealed that loss of ITGA5 inhibited growth of peritoneal OC xenografts over time (Fig. 2, L and M). Collectively, these data suggest that the ITGA5^high^ cells in the ascites are crucial for peritoneal adhesion and metastasis.

### ATCs form heterotypic spheroids, named MUs, with CAFs in HGSOC and discriminate from LGSOC

**Figure 3.**
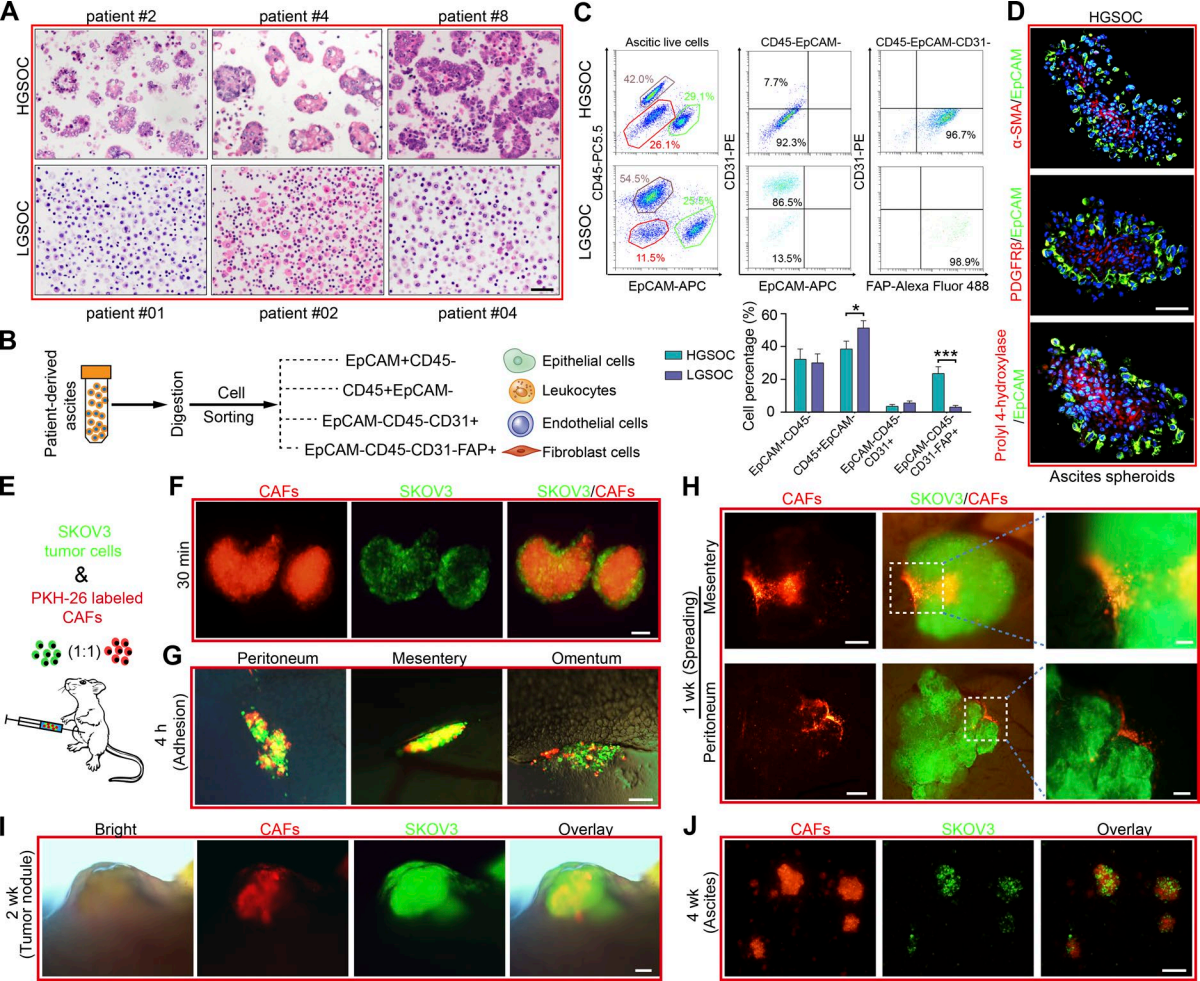
**CAF-centered heterotypic spheroids act as the MU during OC peritoneal dissemination. (A)** Representative H&E staining of ascites tissues collected from HGSOC (#2, 4, and 8) and LGSOC patients (#01, 02, and 04). Bar, 50 µm. **(B)** The single cells from ascites were subjected to flow cytometry analyses of the percentages of these epithelial cells, leukocytes, endothelial cells, and fibroblasts. **(C)** Representative flow cytometry plots (top) and frequencies (bottom) showing the expression percentage of the above mentioned major resident cell categories in HGSOC and LGSOC ascites. **(D)** Dual immunofluorescence staining for EpCAM combined with α-SMA, PDGFRβ, or prolyl 4-hydroxylase in HGSOC-derived spheroids. Bar, 100 µm. **(E)** Schematic illustration of peritoneal implantation of equivalent GFP-transfected SKOV3 cells and PKH-26 labeled CAFs (#1, 2, and 5). **(F and G)** Representative immunofluorescence image of the peritoneal heterotypic spheroids 30 min (F) and their adhesion to favored locations such as peritoneum, mesentery, and omentum 4 h after initial implantation of tumor cells and CAFs. Bar, 100 µm. **(H)** Immunofluorescence images showing the spreading of adhered spheroids in the peritoneal cavity 1 wk after implantation. Bars, 200 µm (left) and 50 µm (right). **(I)** Representative images of the newly formed metastatic nodules 2 wk after implantation. Bar, 200 µm. **(J)** Representative immunofluorescence images of heterotypic spheroids in murine ascites 4 wk after initial implantation. Bar, 100 µm. Data are means ± SEM and representative of four (A, C, and D) or three (F–J) independent experiments. *, P < 0.05; ***, P < 0.001, determined by Student’s *t* test.

The phenotype and biology of ATCs is shaped by influences from ascites that constitutes their unique resident microenvironment. Malignant ascites is not only a common feature of advanced OC, but it also nourishes and constantly modulates the behavior of disseminated tumor cells which adapt to their new environment to become resident ATCs (Penson et al., 2000; Lane et al., 2011). In the context of ascites fluid collected from HGSOC and LGSOC patients, we observed that the HGSOC ascitic cells combine to form spheroids, whereas the LGSOC ascitic cells are prone to exist in solitary form or as smaller aggregates (Fig. 3 A). Besides tumor epithelial cells (EpCAM^+^; epithelial cell adhesion molecule), CAFs (FAP^+^; fibroblast activation protein), immune cells (CD45^+^), and endothelial cells (CD31^+^) are the main cell types in ascites (Erez et al., 2010). A subsequent compositional analysis identified fibroblasts (EpCAM^−^CD45^−^CD31^−^FAP^+^) as the cellular component showing the largest difference between HGSOC and LGSOC ascites (Fig. 3, B and C). These observations raise the possibility that ascitic fibroblasts might influence ATC behavior and OC progression.

Indeed, immunofluorescence analysis revealed that HGSOC-derived ascitic spheroids contained architectures characterized by EpCAM^+^ epithelial cells surrounding a core of CAFs, characterized by α-smooth muscle actin (α-SMA), platelet-derived growth factor receptor-β (PDGFRβ), or prolyl 4-hydroxylase staining (Fig. 3 D). Therefore, we hypothesized that CAFs participate in ascitic spheroid formation and subsequent transcoelomic metastasis of OC. We established a suspension coculture of SKOV3 and CAFs and found that tumor cells were present in the form of single cells, SKOV3-only spheroids (homospheroids), and SKOV3/CAFs heterotypic spheroids (heterospheroids). Indeed, heterospheroids displayed the strongest adhesive capacity, followed by homospheroids, and then single tumor cells (Fig. S2, A and B). Subsequent anoikis assays revealed that tumor cells in the heterospheroids displayed the lowest apoptosis rate in suspension culture (Fig. S2 C), further supporting that spheroid tumor cells are more resistant and prone to metastasis. Moreover, heterospheroids show enhanced mesothelial clearance, invasion, and spreading capabilities (Fig. S2, D–F).

To further explore the contribution of CAFs and associated heterospheroids during early OC transcoelomic metastasis, we established a mouse model in which PKH-26–labeled CAFs and GFP-transfected SKOV3 were i.p. injected into NOD-SCID female recipient mice (Fig. 3 E). Intriguingly, typical heterospheroids formed instantaneously and adhered to their metastatic tropism omentum (Fig. 3, F and G), followed by spreading of heterospheroids and subsequent development of pronounced protruding tumor nodules with a CAF-derived stroma (Fig. 3, H and I). Consequently, characteristic heterospheroids appeared in murine ascites that had developed at the end of the experiment (Fig. 3 J). We conclude that exogenous CAFs support adhesion and metastasis of tumor cells by forming heterospheroids.

To further demonstrate the involvement of endogenous CAFs in mediating heterotypic spheroid formation and early transcoelomic metastasis, GFP-transfected SKOV3 cells were implanted either i.p. or orthotopically. Heterospheroids could also be observed early after implantation in the murine peritoneal cavity, with GFP^+^ tumor cells surrounding the GFP^−^ stromal cells. Subsequent constitutional analyses revealed that fibroblasts (EpCAM^−^CD45^−^CD31^−^FAP^+^) were the major ingredient of GFP^−^ host stromal cells (Fig. S3, A–D). This phenomenon was further observed in the EGFP transgenic mice after peritoneal injection of PKH-26–labeled syngeneic ID8 tumor cells, which resulted in the development of ascitic spheroids consisting of GFP^+^ stromal cells surrounded by tumor cells. Again, fibroblasts proved to be the major component of the GFP^+^ core population by flow cytometry analyses and immunofluorescence (Fig. S3, E–G). Due to its inherent malignant potential and contribution to peritoneal dissemination, we termed this CAF-centered, compact, heterospheroid structure an MU.

### HGSOC derived CAFs facilitates MU formation and peritoneal metastasis of LGSOC ATCs

**Figure 4.**
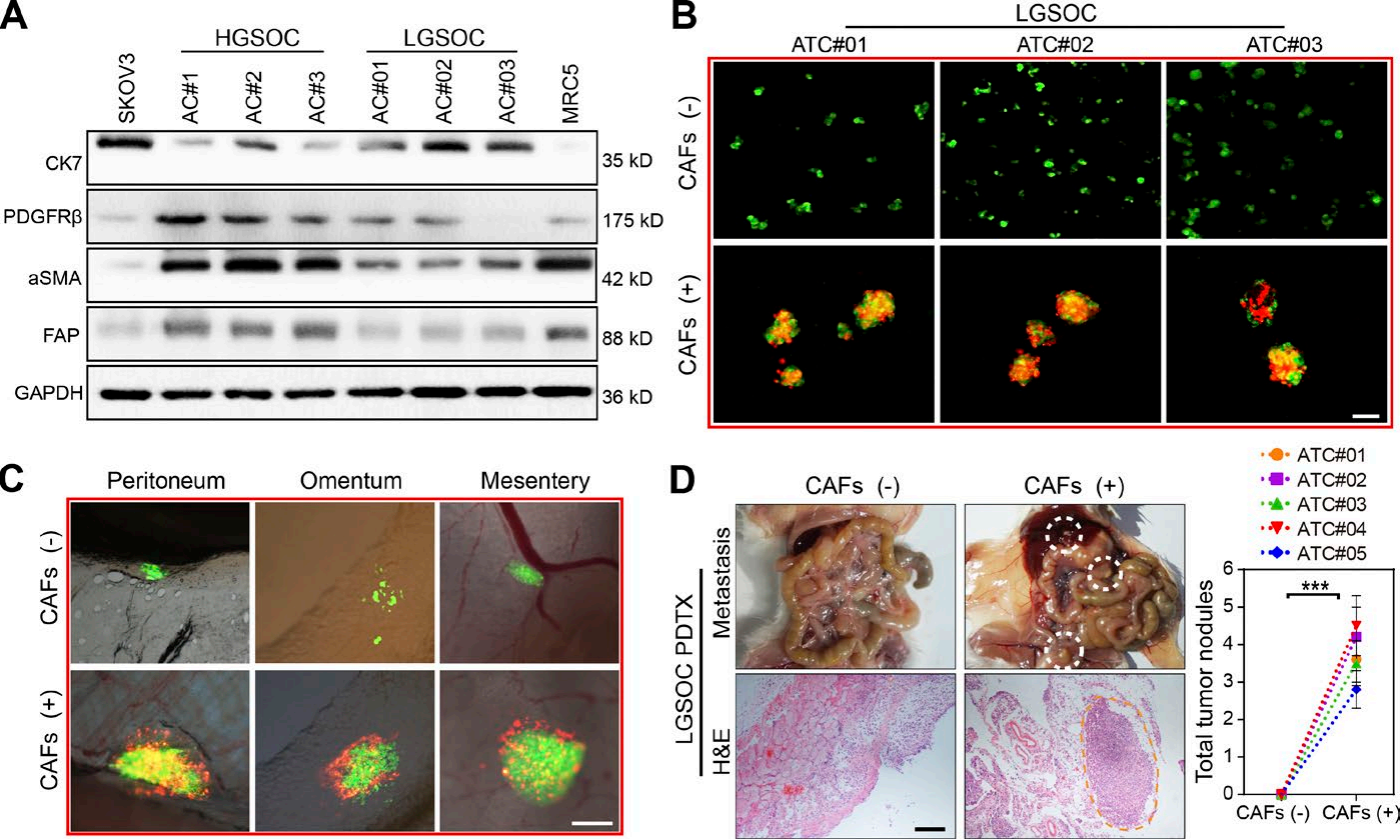
**HGSOC-derived CAFs facilitate MU formation in LGSOC ATCs and promote peritoneal metastasis. (A)** Immunoblotting of CAF markers (FAP, α-SMA, and PDGFRβ) and the epithelial marker CK7 in ascites cells (ACs) from HGSOC (AC#1–3) and LGSOC (AC#01–03) patients. SKOV3 served as the positive control for epithelial cells while MRC5 cells served as the positive control for fibroblasts. **(B)** Representative images of heterotypic spheroids formation by ATCs derived from LGSOC in suspended culture, in the absence or presence of HGSOC-derived CAFs (#2, 3, and 6). Bar, 50 µm. **(C)** Representative images for peritoneal adhesion of LGSOC ATCs (#02) in the absence or presence of CAFs (#2, 4, and 7) derived from HGSOC. Bar, 100 µm. **(D)** Representative images and quantification of peritoneal metastases in mice bearing ATCs derived from LGSOC (#01–05), in the absence or presence of HGSOC-derived CAFs (#2, 7, and 8; *n* = 8 mice per group). Bar, 200 µm. Data are means ± SEM and are representative of two (A, C, and D) or three (B) independent experiments. ***, P < 0.001, determined by Student’s *t* test.

Our previous results had already shown that CAFs are rare in the peritoneal cavity of LGSOC patients, which was further validated by immunoblotting of representative fibroblast activation markers in ascitic cells from HGSOC and LGSOC patients (Fig. 4 A). To evaluate whether exogenous CAFs could alter the biological behavior of LGSOC ATCs, we compared the adhesion and metastatic capacity of them in the absence or presence of HGSOC-derived CAFs. Intriguingly, addition of HGSOC-derived CAFs to ATCs from LGSOC facilitated formation of typical heterotypic spheroids and subsequent peritoneal adhesion (Fig. 4, B and C). Consequently, ATCs derived from LGSOC showed notably enhanced tumor xenograft growth when HGSOC-derived CAFs were present (Fig. 4 D). Thus, the clinical observation of a slower disease progression natural history in LGSOC might be ascribed, in part, to the lack of ascitic CAFs and subsequent MUs.

### ITGA5 mediates the adhesion of ATCs with CAFs in the formation of MUs

**Figure 5.**
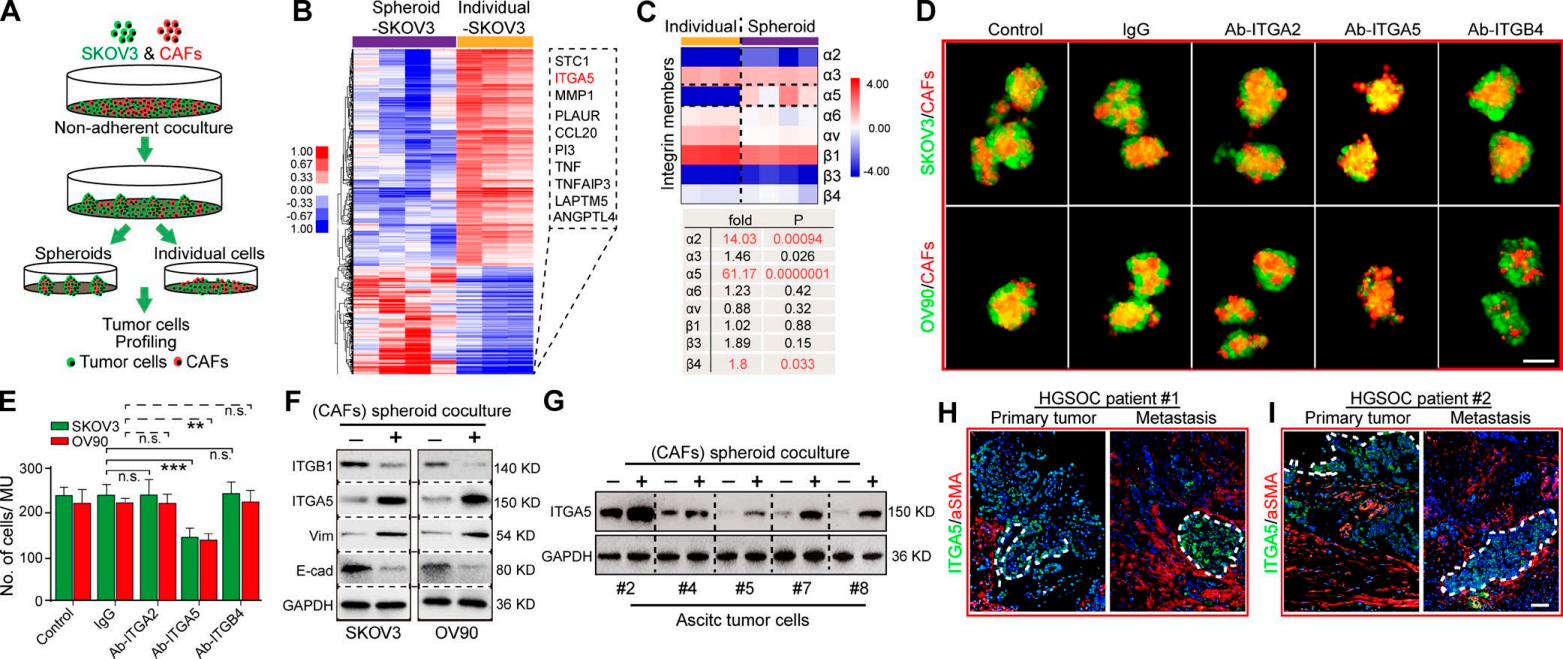
**CAFs selectively recruit ITGA5^high^ ATCs to form MUs and further sustain ITGA5 expression in ATCs. (A)** Drawing depicting the separation of SKOV3 cells forming heterotypic spheroids with CAFs (#1–4) from individual SKOV3 cells in nonadherent culture condition and further steps toward mRNA profiling. **(B)** Heat map detailing the differentially expressed genes between SKOV3 cells isolated from heterotypic spheroids and those remaining individual SKOV3 cells. The top 10 genes overexpressed in spheroid SKOV3 cells are shown in the box (right). **(C)** Comparative analysis of the relevant integrin expression level extracted from the microarray data. **(D)** Representative images of heterotypic spheroids formed by GFP-transfected tumor cells with CAFs (#5, 8, and 9), in the presence or absence of IgG, neutralizing antibody to integrin α2 (Ab-ITGA2), α5 (Ab-ITGA5), or β4 (Ab-ITGB4). Bar, 100 µm. **(E)** The average number of cells included in the spheroids formed from each of the groups described in D was quantified. **(F)** Immunoblotting for E-cadherin, Vim, ITGA5, and ITGB1 in SKOV3 and OV90 cells before and after coculture with CAFs (#7 and 8) in heterospheroids. **(G)** Immunoblot validation of ITGA5 expression alteration in primary ATCs before and after coculture with CAFs (#7 and 8) in heterospheroids. **(H and I)** Representative images of dual immunofluorescence staining of ITGA5 and α-SMA in primary tumor and metastases in two cases of HGSOC patients, counterstained with DAPI. Bar, 50 µm. Data are means ± SEM. Data are one experiment (B and C) and representative of three (D and E) or two (F–I) independent experiments. **, P < 0.01; ***, P < 0.001; n.s., no significance; determined by Student’s *t* test.

We reasoned that adhesion of tumor cells to CAFs is required for formation of MUs. As integrin family members are known to mediate tumor cell adhesion in spheroids (Leroy-Dudal et al., 2005; Suzuki et al., 2005; Santiago-Medina and Yang, 2016), we compared the expression pattern of tumor cells that could form MUs with CAFs and those remaining individual tumor cells. SKOV3 in spheroids and individual SKOV3 tumor cells were thus isolated and subjected to molecular profiling (Fig. 5 A). Significantly, ITGA5 was identified as the second-most elevated gene in spheroid-SKOV3 compared with individual SKOV3 cells (Fig. 5 B; Table S4). Among the integrin family members, integrin α2, α5 and β4 were significantly higher in spheroid-SKOV3 compared with individual SKOV3 cells (Fig. 5 C). To specifically assess the involvement of the aforementioned integrins in MU formation, we employed specific neutralizing antibodies to block them and found that blocking of ITGA5 could drastically blunt MU generation by CAFs with SKOV3 and OV90 cells (Fig. 5, D and E).

In addition, tumor cells exhibited elevated ITGA5 and VIM levels and reduced integrin β1 (ITGB1) and CDH1 levels after suspended coculture with CAFs in MUs, thus indicating that interaction with CAFs in MUs could further maintain the ITGA5^high^ mesenchymal phenotype in ATCs (Fig. 5 F). Immunoblotting in several primary ATCs further revealed that ITGA5 was notably up-regulated after coculture in MUs with CAFs (Fig. 5 G). In the context of OC tumor tissues, ITGA5 staining was predominantly detected at the invasive front of HGSOC specimens and areas adjacent to stromal CAFs (Fig. 5, H and I). These data indicate that the fibroblast backbone may selectively attract ITGA5^high^ ATCs to form MUs, in which ITGA5 expression by ATCs is then maintained.

#### EGF derived from CAFs within MUs is required to maintain ITGA5 expression in ATCs

**Figure 6.**
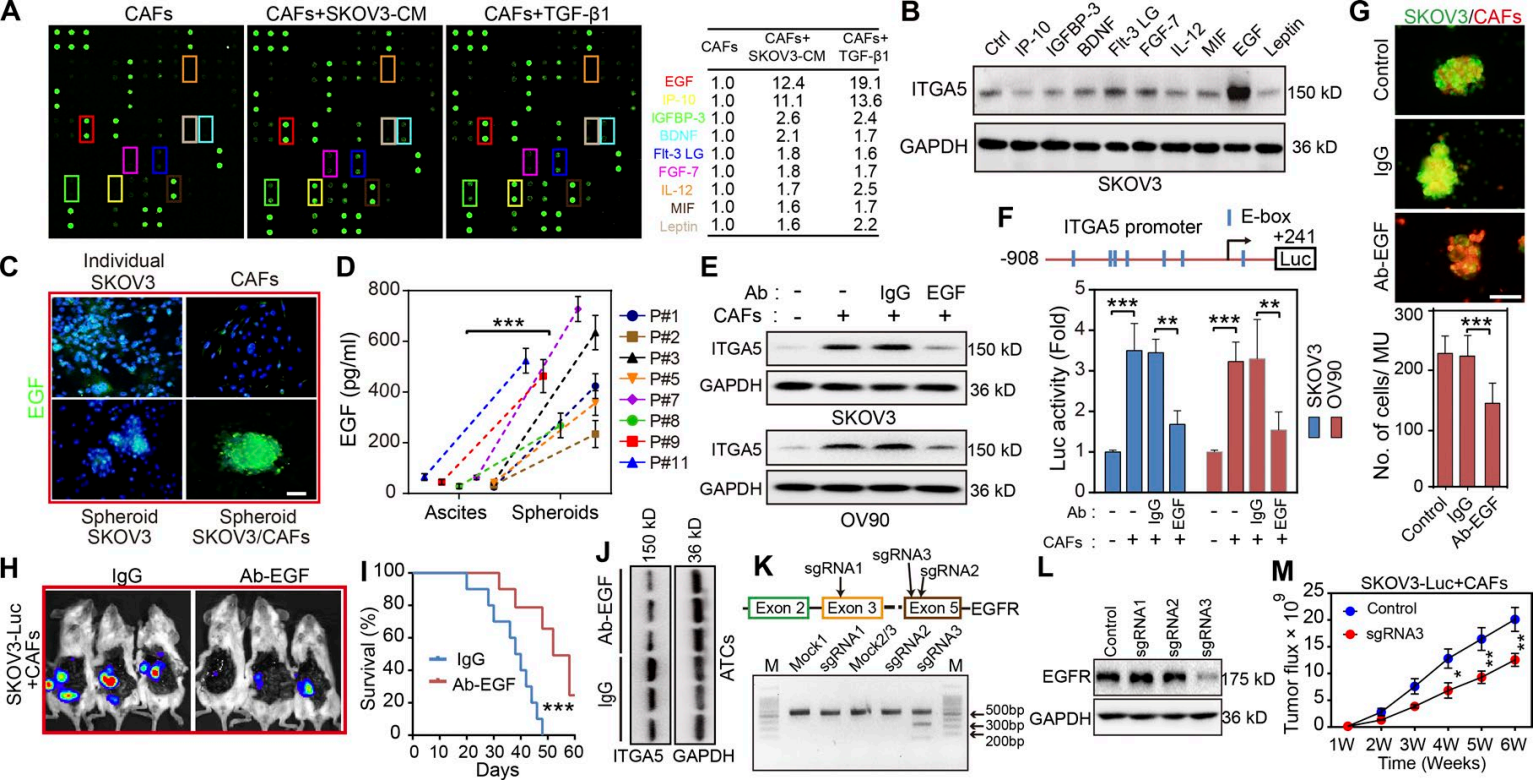
**CAF-dependent EGF secretion is required for ATC ITGA5 expression in MUs. (A)** Cytokine profile from control CAFs (#1), and from CAFs primed either with SKOV3 CM or with TGF-β1 (100 ng ml^–1^) for 48 h. The shared cytokines elevated in CAFs treated with CM or TGF-β1 are listed. **(B)** Western blot analysis of ITGA5 in SKOV3 cells treated with variant cytokines for 48 h. **(C)** Representative immunofluorescence images of EGF in SKOV3 and CAFs (#8 and 9) in adherent culture, in SKOV3 homospheroids, and in MUs formed by SKOV3 and CAFs. Bar, 50 µm. **(D)** EGF secretion in the MU microenvironment and in the corresponding ascites macroenvironment of eight HGSOC patients was assessed by ELISA. **(E)** Immunoblotting for ITGA5 in control and in isolated tumor cells following coculture with CAFs (#6 and 8) in MUs, in the presence or absence of either EGF-neutralizing antibody or IgG control. **(F)** Schematic representation of the *ITGA5* promoter reporter constructs (top) and analysis of *ITGA5* promoter activity (bottom) in variant tumor cell groups, as described in E. **(G)** Representative images and quantification of heterotypic spheroid formation by SKOV3 and CAFs (#4, 9, and 10), in the presence or absence of IgG or EGF-neutralizing antibody. Bar, 100 µm. **(H and I)** Representative i.p. bioluminescence images (H) and survival curves (I) for mice bearing tumors generated by coinjection of SKOV3-Luc and CAFs (#7 and 9) in the control IgG and the EGF-neutralizing antibody group (*n* = 10 mice per group). **(J)** Immunoblot of ITGA5 in peritoneal tumor cells isolated from the groups treated with either control IgG or EGF-neutralizing antibody. **(K)** Editing EGFR in SKOV3 using the CRISPR/Cas9 system. Top: Schematic diagram of sgRNA1-3 targeting exon 3/5 of the EGFR gene. Bottom: The genomic PCR products of SKOV3 cells transduced with scrambled sgRNA (mock) or sgRNA1-3 were analyzed with T7E1 assay. **(L)** SKOV3 cells transduced with scrambled sgRNA (control) or sgRNA1-3 were subjected to immunoblotting with EGFR. **(M)** Tumor growth curves developed by control and EGFR-deficient SKOV3-Luc cells in combination with CAFs (#9 and 10; *n* = 10 mice per group). Data are means ± SEM and representative of two (A, B, E, and H–M) or three (C, D, F, and G) independent experiments. *, P < 0.05; **, P < 0.01; ***, P < 0.001, determined by Student’s *t* test.

Numerous studies have revealed that interactions between tumor and stroma depend on cytokines (Orimo et al., 2005; Chen et al., 2014). To identify the cytokines secreted by activated fibroblasts that sustain ITGA5 expression in ATCs, we evaluated the cytokine profiles of conditioned medium (CM) from control CAFs and from CAFs activated by ITGA5^high^ SKOV3–derived CM or by transforming growth factor β (TGF-β1), which was introduced as an inducer or positive control of fibroblast activation. This revealed nine cytokines (EGF, IP-10, IGFBP-3, BDNF, Flt-3 LG, FGF-7, IL-12, MIF, and leptin) that were up-regulated in both groups (Fig. 6 A and Table S5). Among them, EGF displayed the highest correlation with ITGA5 in SKOV3 tumor cells (Fig. 6 B). Moreover, EGF expression in CAFs increased following addition of OC cell CM or TGF-β1 and was suppressed in the presence of TGF-β1–neutralizing antibody (Fig. S4, A and B). Interestingly, immunofluorescence revealed amplified EGF expression in heterospheroids, which was not readily apparent in homospheroids, solitary tumor cells, or CAFs (Fig. 6 C). Accordingly, EGF was much more prevalent in the MU microenvironment compared with the corresponding ascites macroenvironment of HGSOC patients (Fig. 6 D). The importance of EGF for increased ITGA5 expression in tumor cells was confirmed, since addition of an EGF-neutralizing antibody to the suspension coculture attenuated ITGA5 expression in MUs (Fig. 6 E). Reporter assays also demonstrated that *ITGA5* promoter activity was inhibited tremendously in the presence of EGF-neutralizing antibody in spheroid coculture (Fig. 6 F).

Immunostaining in a series of HGSOC tissues revealed that EGF was evenly distributed between epithelial and stromal regions, whereas EGFR was predominantly located in epithelial cells (Fig. S4, C and D). Quantitative PCR analysis in paired tumor cells and CAFs confirmed the above expression pattern of EGF and EGFR (Fig. S4 E), suggesting that EGF in OC microenvironment mainly acts on cancer cells where it helps to maintain their ITGA5^high^ phenotype. Accordingly, neutralizing anti-EGF antibody blunted the formation of MUs by SKOV3 and CAFs (Fig. 6 G). Finally, the EGF neutralizing antibody attenuated peritoneal tumor burden and increased survival in mice following peritoneal injection of SKOV3-Luc cells and CAFs (Fig. 6, H and I), accompanied by diminished ITGA5 expression in ATCs and reduced metastases in the anti-EGF treatment group compared with the IgG group (Fig. 6 J). To further ascertain the role of EGF/EGFR signaling in OC peritoneal dissemination, we used CRISPR/Cas9 editing to knock down EGFR in OC tumor cells. Ablation of EGFR was most evident in tumor cells edited with sgRNA3, confirmed with T7E1 assay and Western blot (Fig. 6, K and L). Tumor growth curves indicated that EGFR-deficient tumor cells showed notably diminished growth rate than EGFR complete SKOV3-Luc coinjected with CAFs (Fig. 6 M). These data suggest that EGF derived from activated fibroblasts are amplified inside MUs and promote spheroid formation and ITGA5 expression in attached tumor cells.

#### Disruption of MU integrity by targeting CAFs attenuated early-stage peritoneal metastasis

**Figure 7.**
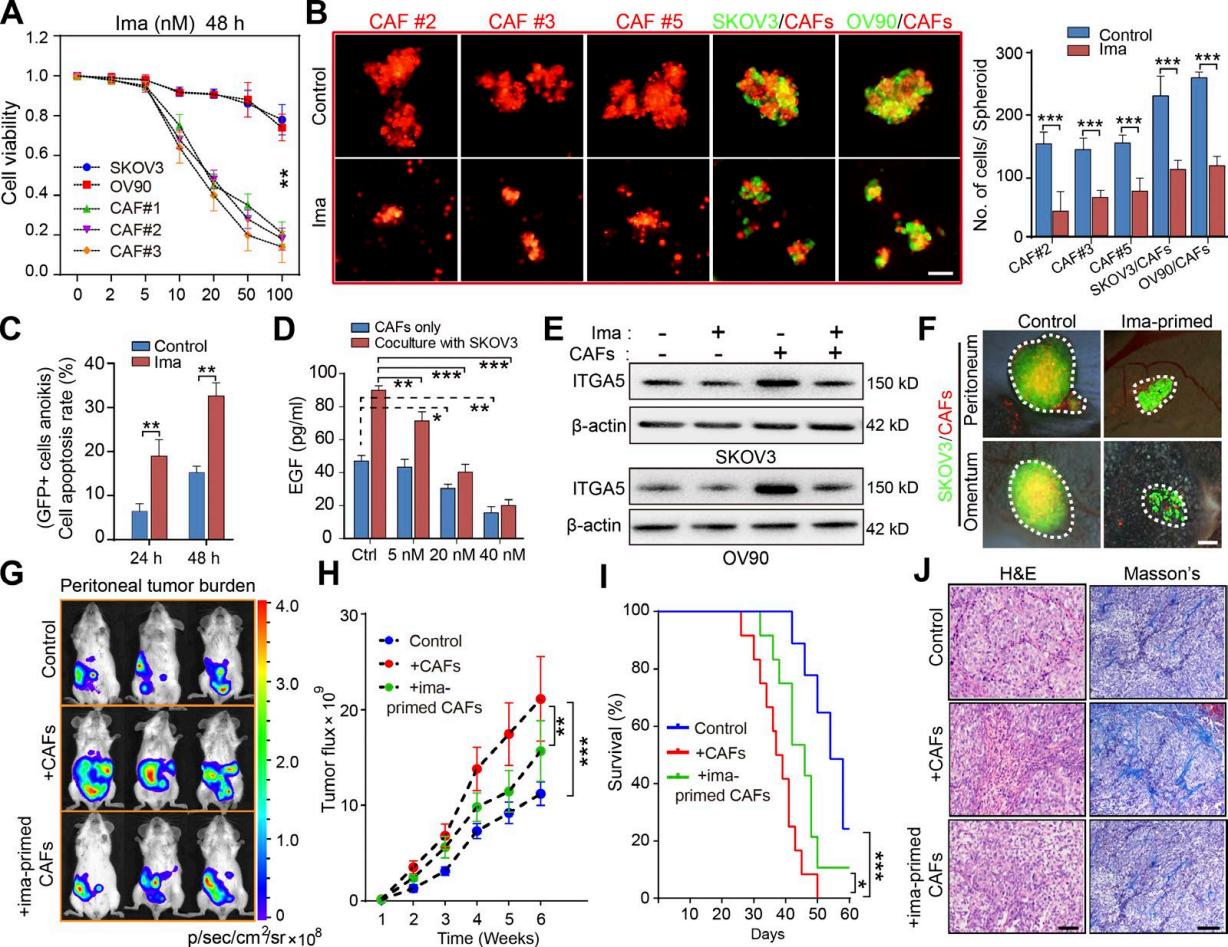
**Targeting CAFs in ascites disrupts MUs and attenuates OC dissemination. (A)** Cell viability analysis of the OC tumor cells and of primary CAFs (#1–3) exposed for 48 h to varying doses of imatinib (ima; 0–100 nM). **(B)** Representative images and quantification of spheroid formation by primary CAFs (#2, 3, and 5) or in suspended coculture with GFP-transfected tumor cells, in the presence or absence of 20 nM imatinib. Bar, 50 µm. **(C)** Flow cytometry analysis of cellular apoptosis rate for GFP^+^ SKOV3 cells cultured in heterotypic spheroids with CAFs (#2, 5, and 6) for the indicated time, in the presence or absence of 20 nM imatinib. **(D)** EGF secretion by CAFs (#3, 5, and 8) cocultured or not with SKOV3 cells, in the presence or absence of varying doses of imatinib, was assessed by ELISA. **(E)** Immunoblot of ITGA5 in control SKOV3 and OV90 cells, or after heterotypic coculture with CAFs (#5 and 8), in the presence or absence of 20 nM imatinib. **(F)** Representative images of peritoneal sphere adhesion 1 wk after coimplantation of SKOV3 cells with ima-primed CAFs (#3, 7, and 8) or untreated controls. Bar, 50 µm. **(G–I)** Representative bioluminescence images (G), tumor growth curves (H), and survival curves (I) in SKOV3-Luc tumor-bearing mice coimplanted with imatinib-primed CAFs (#7 and 8) or untreated controls (*n* = 10 mice per group). **(J)** H&E and Masson’s trichrome staining of tumors from mice implanted with SKOV3-Luc cells only or coimplanted with ima-primed or untreated CAFs. Bars, 50 µm (left) and 100 µm (right). Data are means ± SEM and representative of four (A–C), two (D, E, and G–J) or three (F) independent experiments. *, P < 0.05; **, P < 0.01; ***, P < 0.001; determined by Student’s *t* test.

Experiments performed so far highlighted that the CAF backbone inside a MU recruits and carries ATCs to remote metastatic sites in HGSOC patients, raising the possibility that therapeutic interventions targeting CAFs could disrupt MU integrity and thus hamper metastatic implantation in the peritoneum and beyond. Imatinib—a small-molecule tyrosine kinase inhibitor with activity against BCR-ABL, c-KIT, and PDGFR—was selected on the basis that PDGF signaling is important for CAF survival (Murata et al., 2011). Enhanced PDGFR expression in stromal fibroblasts (Fig. S5 A) provided a further rationale. At concentrations <20 nM, imatinib selectively suppressed OC primary CAF viability and had a negligible effect on OC cell, macrophage, and endothelial cell survival (Fig. 7 A and Fig. S5 B). As expected, the addition of imatinib completely destroyed the central CAF skeleton and thus prevented subsequent heterospheroid formation by CAFs and OC cells (Fig. 7 B). In addition, imatinib increased the apoptosis rate of GFP^+^ tumor cell in coculture with CAFs in MUs (Fig. 7 C). Moreover, basal EGF secretion by CAFs and in the CAF/tumor cell coculture system was dose-dependently decreased by imatinib (Fig. 7 D). Up-regulation of ITGA5 in tumor cells after coculture with CAFs in heterospheroids was thus attenuated in the presence of imatinib (Fig. 7 E).

With imatinib’s blunting of CAF activity and their interaction with tumor cells, we observed a sharp reduction in peritoneal adhesion of MUs when CAFs were treated with imatinib before interacting with SKOV3 cells (Fig. 7 F). Finally, pretreatment with imatinib significantly reduced the role of exogenous CAFs in facilitating peritoneal metastasis and improved survival of mice bearing SKOV3-Luc peritoneal xenografts (Fig. 7, G–I). To further ascertain the ability of imatinib to interfere with tumor-promoting effects of endogenous activated fibroblasts in OC xenografts, we developed an orthotopic mouse model to explore different schemes of imatinib administration (Fig. S5 C). This revealed that only early-stage imatinib intervention was sufficient to decrease peritoneal tumor burden and improve mouse survival. Primary tumor growth, however, remained unaffected by imatinib in the SKOV3-Luc orthotopic model (Fig. S5, D–F). Both H&E and Masson trichrome staining revealed that tumors arising from the imatinib-pretreated and early-phase imatinib intervention groups displayed a less stroma-rich architecture (Fig. 7 J and Fig. S5 G).

Moreover, host immune cells, especially TAMs, were reported to interact with CAFs and drive spheroid formation in OC (Yin et al., 2016), which was supported by our finding that CD45^+^ immune cells appeared together with MUs developed in xenograft models. Therefore, we extended our research to evaluate depletion of TAM in ascitic microenvironment influence of OC dissemination, using liposome clodronate (LC) alone or in combination with imatinib in a BALB/c nude mouse xenograft model. Removal of macrophages via LC significantly retarded peritoneal tumor metastasis, reinforced the tumor-suppression role of imatinib, and thus improved mouse survival bearing orthotopic OC xenograft (Fig. S5, H–J). The above findings emphasized the nonnegligible contribution of TAM in OC peritoneal dissemination besides CAFs. Collectively, these results demonstrate that targeting CAFs could disrupt metastasis-prone MUs, thus interfering with CAF-dependent tumor cell adhesion and OC dissemination.

## Discussion

**Figure 8.**
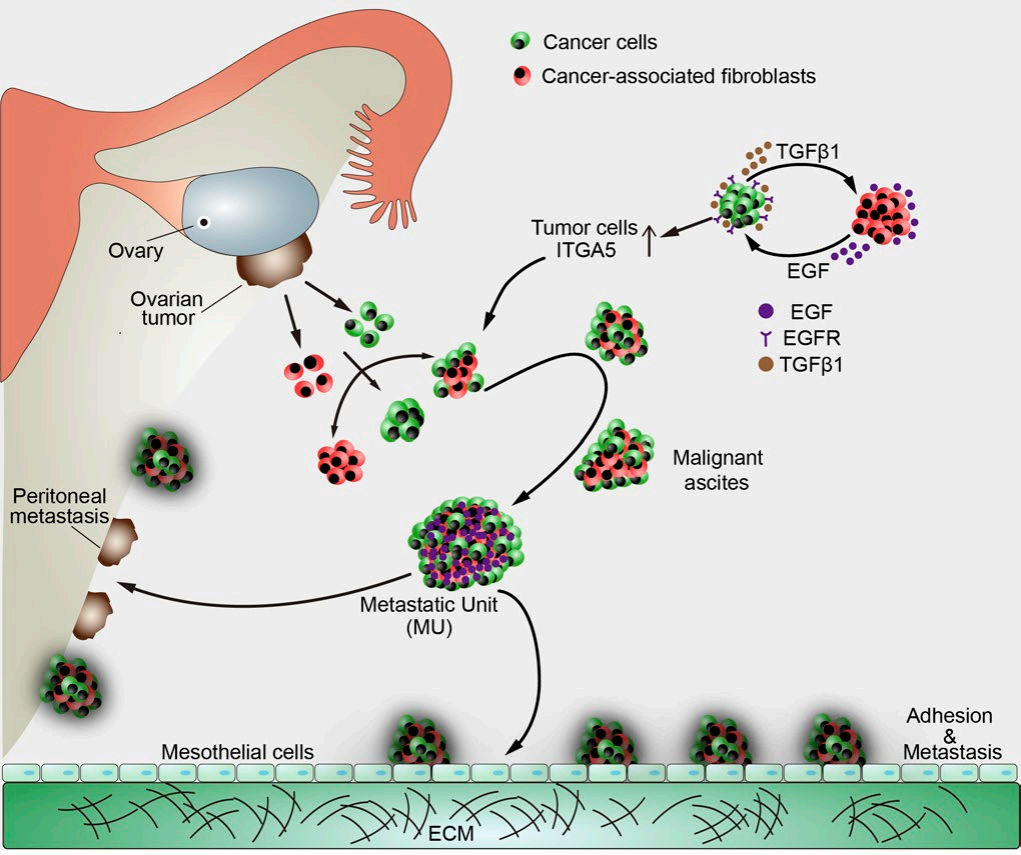
**Model of the development of CAF-centered MUs and their role in OC peritoneal dissemination.** During early stages of OC transcoelomic metastasis, tumor cells detach from the primary tumor and meet with activated fibroblasts in the peritoneal cavity. Interactions between tumor cells and CAFs generate specialized compact heterotypic spheroids, which we named MUs of OC for their pivotal role in peritoneal adhesion and invasion. ITGA5 mediates ATCs interaction with CAFs located in the center of MUs, where they provide initial matrix support for OC to avoid anoikis. Importantly, activated CAF-secreted EGF is enriched within the MU architecture and specifically induces enhanced ITGA5 expression on ATCs, thus strengthening the integrity of MUs and ultimately promoting exacerbation of OC.

A detailed understanding of how ATCs contribute to further metastasis is crucial to better comprehend the biological complexity of HGSOC. In this study, we defined an OC MU characterized by a CAF skeleton surrounded by tumor cells, which was prevalent in HGSOC ascites and actively involved in peritoneal dissemination. ITGA5^high^ ATCs were selectively recruited by CAFs to form the unique heterotypic spheroids. CAFs thus support ATC survival, guide their further peritoneal and transperitoneal adhesion and invasion, and finally constitute the tumor stroma in newly formed metastases. Moreover, EGF derived from fibroblasts under ATCs stimulation was significantly enriched within MUs, where it increases ITGA5 expression in ATCs, thereby further strengthening interactions between ATCs and CAFs (Fig. 8). Targeting CAFs could destroy MU integrity and limit peritoneal tumor cell implantation in an OC xenograft model. Thus, we provide mechanistic and clinical insight into the role of spheroid-associated CAFs in human OC progression.

The supporting role of stromal elements during nidation and expansion of metastatic colonies has been well recognized. Interestingly, a novel powerful mechanism including paracrine lipid supply of tumor cells from omental stromal cells has recently been described (Nieman et al., 2011). Here, we further expand the prometastatic function of the stroma to the very early steps of metastatic spread. Given its substantial population of malignant ATCs, as well as the presence of various recruited and transformed host cells, malignant ascites in OC should not merely be seen as a fluidic environment but rather as dispersed tumor tissue. Interestingly, stromal fibroblasts were abundant in ascites and are prone to associate with ATCs to form prometastatic MUs in HGSOC. The aggressive form of heterotypic spheroids identified in our experiments differs from previously reported multicellular aggregates in ascites by comprising not merely tumor cells. The heterotypic spheroids that we identified as MUs are more similar to recently discovered heterotypic spheroids with TAMs at their center (Yin et al., 2016). Both findings emphasize the contribution of ascitic nonepithelial cellular components to spheroid formation and subsequent dissemination of OC. Our results also uncovered the synergistic effect of simultaneous targeting CAFs and TAMs in hampering OC peritoneal metastasis. Analogously, partial depletion of CAFs significantly decreased the number of metastases in a lung metastatic model (Duda et al., 2010). Fibroblast-associated tumor cell cluster facilitation of tumor growth was also observed in pancreatic ductal adenocarcinoma, ampullary adenocarcinoma, and cholangiocarcinoma (Arnoletti et al., 2018). Intriguingly, stromal fibroblasts and the resultant MU structure were rarely found in LGSOC ascites, which might explain its much lesser tendency for dissemination and implantation. Subsequently, we observed that exogenous HGSOC-derived CAFs promote MU formation and fuel peritoneal metastasis of LGSOC ATCs. Based on these findings, these highly aggressive CAF-centered MUs appear to be essential to OC dissemination and might thus substantially contribute to the biological differences between HGSOC and LGSOC.

The role of early peritoneal adhesion in OC abdominal metastasis and the notion that exposure to stromal cells can enforce metastatic behavior in neoplastic epithelial cells has increasingly been realized over recent years (Nieman et al., 2011; Pankova et al., 2016). Interestingly, a recent study highlighted that fibroblasts recruited to solid ovarian tumors could be used to detect insidious tumor lesions (Oren et al., 2016). However, stromal fibroblasts already appear in the peritoneal cavity already soon after tumorigenesis, where they recruit exfoliated tumor cells to form MUs, which then adhere and metastasize. Because the PDGFR inhibitor imatinib selectively eradicates fibroblasts and thus disrupts the integrity of MUs, early administration of imatinib dramatically attenuates peritoneal implantation. At a more advanced stage of disease, however, imatinib showed only negligible effects in our murine metastasis model. This highlights that MU-driven adhesion occurs early during the metastatic cascade leading to OC dissemination. Unfortunately, clinical trials on the use of imatinib in OC were performed on patients with platinum-resistant, multiple drug–exposed recurrent OC, which likely explains the dismal outcome (Juretzka et al., 2008; Matei et al., 2008). In light of our data, trials on the use of imatinib in OC should be designed with a low dosage targeting CAFs in the early stage of the disease or in patients who had achieved complete cytoreduction after primary debulking surgery, or should be suitable for maintenance therapy after the salvage chemotherapies such as bevacizumab or olaparib. Thus, clinical data are fully compatible with the presumed role of ascitic CAFs in OC metastasis. Early eradication of CAFs thus appears critical for the effective targeting of MUs.

The detailed mechanism underlying spheroid formation between tumor cells or between tumor cells and other ascitic cells was, however, largely unknown. It had been reported that free tumor cells detached from the primary tumor might form spheroids through interactions between α5β1 integrin and fibronectin (Santiago-Medina and Yang, 2016). In the present study, we found that stromal fibroblasts selectively recruited ITGA5^high^ ATCs. They are thought to initially originate from detached tumor cells that lost their E-cadherin during tumor initiation and progression (Sawada et al., 2008). The inverse correlation between ITGA5 and E-cadherin was reported previously (Sawada et al., 2008) and observed in our study. In addition, tumor cells in the invasive front of primary tumors were demonstrated in our study to present ITGA5^high^ phenotype, which were more prone to drop into the peritoneal cavity under gravity or intrinsic mechanisms. In this scenario, we therefore ascribed the lack of spheroid formation in LGSOC to the lack of ascitic fibroblasts, rather than the shortage of ITGA5^high^ ATCs. Blocking ITGA5 drastically attenuates spheroid formation between tumor cells and fibroblasts. Moreover, ITGA5 expression correlates with dismal patient outcome in various cancer types (McKenzie et al., 2013; Ren et al., 2014; Santiago-Medina and Yang, 2016). However, recent clinical trials involving monotherapy with a neutralizing antibody against ITGA5 failed to demonstrate benefit in HGSOC patients (Ricart et al., 2008; Bell-McGuinn et al., 2011). This finding suggests that ITGA5 blockage hampers early MU formation, whereas it might not be effective when the metastatization process is already established. Mechanistically, we demonstrated that tumor cells promote EGF secretion by CAFs within the MU microenvironment that consequently drives ITGA5 expression in ATCs, which in turn further strengthens the tumor–stromal interaction inside MUs. Besides EGF acting as a soluble factor, the increase in EGF-mediated, increased cell–cell contact might also contribute to tumor cell ITGA5 expression through cell-to-cell contact–dependent factors as described previously concerning other integrins (Chandrasekaran et al., 2000; Masszi et al., 2004; Kim et al., 2009b). Early addition of a neutralizing anti-EGF antibody consequently delayed formation of MUs, prevented ITGA5 increase in ATCs, and attenuated peritoneal metastasis in OC xenografts. In parallel, a recent study demonstrated that inhibition of the EGF/EGFR signaling axis reduced spheroid formation between ATCs and macrophages, and eventually attenuated OC tumor growth in a mouse model (Yin et al., 2016). These data not only emphasize the indispensable role of accessory cells such as CAFs in OC metastasis; they also indicate that EGF represents an attractive alternative therapeutic target to interfere with OC dissemination. Possible effects of EGF blockade on normal host cells will have to be explored.

Altogether, this study highlights the unique aggressive nature of the previously underappreciated ITGA5^high^ ATCs. Stromal CAFs recruit ATCs to form unique compact heterotypic spheroids capable of peritoneal adhesion and metastasis. Our findings thus underscore the pivotal role of MUs in malignant ascites for peritoneal dissemination and define their presence as a major characteristic to discriminate between HGSOC and LGSOC. Furthermore, approaches for the early targeting of stromal CAFs to destroy MUs emerge as new therapeutic strategies to limit HGSOC progression.

## Materials and methods

### Magnetic sorting of tumor cells and fibroblasts from patient-derived tumor tissues

Human tumor samples were obtained from patients diagnosed with advanced stage of HGSOC and LGSOC who had not received preoperative chemotherapy and for some of whom matched primary tumor, ascites (abdominal washings in LGSOC), and metastases tissues were available (Table S6). All patient tissues were collected from the Department of Gynecological Oncology (Tongji Hospital, Huazhong University of Science and Technology, Wuhan, China) between March 2012 and July 2016 with the informed consent of patients and the authorization of the Ethics Committee of Tongji Hospital and assessed by two senior pathologists. Epithelial tumor cells in tumor tissues of various origins were isolated by the magnetic bead sorting (MACS) system (Miltenyi Biotech). Briefly, for primary and metastatic tumors, single-cell suspensions were obtained by enzymatic dissociation of small pieces of fresh tumor excision (∼1 mm^3^) with 1 mg/ml collagenase and 2 mg/ml hyaluronidase (Sigma) for ∼1–2.5 h at 37°C on an orbital shaker. Digested tumor cellular morphology and viability were checked with calcein (green) and ethidium homodimer (red; Thermo Fisher Scientific) staining every 20 min to avoid inadequate or excessive digestion. Enzymatic reaction was terminated by adding 10% FBS. For ascites samples, mild centrifugation (120 *g*, 6 min) was performed to obtain concentrated ascites. Then, all the tumor samples were subjected to RBC lysis buffer (BioLegend) and washed twice with PBS. The cell suspensions were subjected to the following magnetic sorting procedure after brief disaggregation by trypsinization with diluted 0.125% trypsin (Thermo Fisher Scientific; ∼5 min). Single cells were collected by filtering through cell strainer of 40 µm (BD Biosciences) and then incubated with microbeads conjugated to CD326 (Miltenyi Biotech; 130–061-101) for tumor epithelial cell isolation and to anti-fibroblast microbeads (Miltenyi Biotech; 130–050-601) for fibroblast isolation. After 15 min of incubation on ice, the samples were washed twice with ice-cold PBS and sorted with a MACS column (Miltenyi Biotec). The quality of sorting was determined by flow cytometry with a CD326-FITC antibody (Miltenyi Biotech; 130–110-998) for the epithelial component and FAP-Alexa Fluor 488 (R&D Systems; FAB3715G), PDGFRβ-PE (BioLegend; 323606), and α-SMA antibodies (Abcam; ab7817) for the fibroblast component. Only validated epithelial and fibroblast samples were used further for in vitro and in vivo studies.

### Cell culture

Human OC cell lines SKOV3 and OV90 were purchased from ATCC. Human fibroblast cell line MRC-5 was obtained from the cell bank of the Chinese Academy of Sciences. The human peritoneal mesothelial cell line HMrSV5 was obtained from Jennio Biological Technology. ID8 cells were a gift from the University of Kansas Medical Center. All cell lines were routinely checked for mycoplasma contamination (Lonza) and were authenticated by their source organizations before purchase. All cancer cell lines were maintained in McCoy’s 5A medium containing 10% FBS and 1% penicillin/streptomycin (Thermo Fisher Scientific). MRC-5, HMrSV5, and primary ovarian CAFs were cultured in DMEM/F-12 medium containing 10% FBS and 1% penicillin/streptomycin, at 37°C in a 5% CO_2_ and 80% humidity incubator. SKOV3 cells had been stably transduced with CMV-Fluc-IRES-RFP lentiviral particles (GeneChem) and designated as SKOV3-Luc, which were further used in animal live imaging experiments.

### In vitro and in vivo adhesion

In vitro adhesion assays were performed with primary OC cells transiently stained with PKH-67 (Sigma). 2 × 10^4^ labeled cells were added into each well coated with 10 µg/ml fibronectin or 10 µg/ml collagen (Merck) and incubated for up to 30 min at 37°C. Nonadherent cells were removed by gentle washing with PBS. The remaining cells were counted. The results were presented as proportion of adherent cells based on the total initial cell number. Mouse in vivo adhesion assays were performed in the following way: 2 × 10^6^ PKH-67 labeled tumor cells were injected into the peritoneal cavity of female NOD/SCID mice (*n* = 6 per group). 4 h later, mice were sacrificed. Peritoneum, omentum, and mesentery were excised. After gentle washing with PBS to eliminate nonadherent cells, the adherent cell aggregates were observed and photographed under a fluorescence microscope. Five randomly chosen fields were recorded. The excised tissues were digested with NP-40 (Sigma), and the fluorescence signal was read and recorded. In vivo adhesion was quantified based on absorption at 490 nm. In vivo adhesion of matched ATCs and metastatic tumor cells was normalized to adhesion observed with primary tumor cells.

### Mesothelial clearance assay

Mesothelial clearance was assessed based on a method described previously (Kenny et al., 2014). Fluorescently labeled HMrSV5 mesothelial cells (blue, CMAC; Thermo Fisher Scientific) were plated on a 6-well tissue culture plate and maintained in DMEM/F-12 medium without FBS, supplemented with 5% BSA (Sigma), until confluent (48 h after plating). 1 × 10^6^ PKH-67–labeled primary tumor cells from either HGSOC or LGSOC patients were added to separate wells with confluent mesothelial cells, allowed to attach, and imaged at 36 h under a fluorescence microscope (Olympus). To generate heterospheroids and homospheroids, 4 × 10^5^ GFP-transfected OC cells were mixed or not with equivalent numbers of PKH-26–labeled primary CAFs and suspended in complete culture medium on ultra-low attachment plates (Corning). Spheroids for a subsequent mesothelial clearance test were collected 24–36 h later. To quantify mesothelial clearance, the area covered with GFP-fluorescent spheroids within the blue mesothelial monolayer was measured over time. The data are presented as relative clearance size to that of the control group.

### Patient-derived tumor xenograft model

After magnetic sorting of epithelial tumor cells from HGSOC tumor samples (primary tumors, ascites fluid, and metastases) and confirmation of the purity of the epithelial component, equivalent numbers of freshly isolated tumor cells (2 × 10^7^) from each group were injected i.p. into NOD/SCID mice (*n* = 6 per group). After ∼8 wk, mice in each group were anesthetized, and micrometastatic nodules were examined throughout the abdominal cavity under the microscope. Furthermore, H&E staining was performed to investigate the formation of invisible xenografts in each group.

### FA formation and cell spreading

Freshly isolated tumor cells derived from primary tumors, ascites, and metastases were plated on fibronectin (Merck)-coated coverslips and incubated at 37°C for 2 h in complete DMEM/F-12 medium. Formation of FAs was visualized with an anti-paxillin antibody (Abcam; ab32084), and cytoskeleton alterations were detected using rhodamine phalloidin staining (Thermo Fisher Scientific; R415). Immunofluorescence images were acquired, and ImageJ (National Institutes of Health) was used to quantify cell spreading and FA staining. Images were obtained from three independent experiments and combined together. ≥20 cells from each group were analyzed. The total area of each cell was measured with phalloidin staining. FAs were analyzed by measuring the amount of paxillin staining around the cell periphery and normalizing to the cell area.

### Transwell invasion assay

Invasion assays were performed according to the manufacturer’s instructions (BD Biosciences). Briefly, Transwell chambers with polycarbonate membrane filters (8-µm pore size; Corning Life Sciences) were coated with 20 µl Matrigel (BD Biosciences) solution diluted in McCoy’s 5A medium (vol/vol 1:4). 2 × 10^4^ OC cells were added to the upper compartment. The lower compartment was filled with McCoy’s 5A medium supplemented with 20% FBS. After 48 h of incubation at 37°C, the upper surface of the filter was washed with PBS and cleared of nonmigratory cells with a cotton swab. The remaining cells at the lower surface of the filter were fixed with cold methanol and stained with 0.1% (wt/vol) crystal violet (Sigma). Invasive cells were scored by counting the whole filter with a microscope at ×200 magnification.

### Microarrays and gene expression profiling

After magnetic sorting of tumor epithelial cells from matched primary tumors, ascites and metastases of five HGSOC patients (#1–5) and three LGSOC patients (#01–03), total RNA from these samples was isolated with the RNeasy kit (Qiagen), according to the manufacturer’s protocol. The Human Genome U133 Plus 2.0 Array (Affymetrix) was used for gene expression profiling. Microarray hybridization, washing, staining, and scanning were performed according to standard Affymetrix protocols. The acquired expression data were preprocessed using the MAS5 algorithm to perform background correction and quantile normalization of expression arrays. The random-variance model (RVM) *t* test and *F* test were used for analysis of differentially expressed genes between two or more experimental groups, respectively (Wright and Simon, 2003). All hierarchical clustering was performed using average linkage based on Pearson’s correlation of the log_2_-transformed expression values, and heat maps were displayed with normalized *Z*-scores (Miller et al., 2002). The microarray data were deposited in the National Center for Biotechnology Information Gene Expression Omnibus under accession no. GSE73168.

For comparing the profiles of tumor cells that can or cannot form spheroids with CAFs, we isolated heterotypic spheroids immediately (∼4 h) after the development of spheroids by SKOV3 and primary CAFs derived from HGSOC patients (#1–4) in suspension coculture. Next, magnetic sorting was performed in spheroids and individual cells to separate SKOV3 cells, and then subjected to mRNA profiling. Total RNA extracted from spheroid-SKOV3 cells and individual SKOV3 cells was subjected to gene expression profiling with the Human Transcriptome Array 2.0 (HTA2.0) GeneChip. Data normalization and exploring of differentially expressed genes were performed via the Gene-Cloud of Biotechnology Information online tool. The microarray data were deposited in the National Center for Biotechnology Information Gene Expression Omnibus under accession no. GSE98154.

### Public database analysis

For analyzing the correlation between ITGA5 expression and the epithelial marker CDH1 in OC, the normalized gene expression data from NCI-60 tumor cells collection (http://discover.nci.nih.gov/cellminer/) and TCGA) datasets were obtained, and Pearson correlation analyses were performed between ITGA5 and CDH1. The prognostic significance of ITGA5 was evaluated by performing a meta-analysis of 2,970 epithelial OC patient expression profiles using the “curatedOvarianData” Bioconductor package. Survival curves were calculated using the Kaplan–Meier method, conducted with the R Bioconductor “survival” package.

### Kaplan–Meier analysis

ITGA5 expression was detected in our panel of 160 HGSOC patients based on the available clinical data including time of diagnosis, death, or last follow-up. Overall survival was defined as time from diagnosis to death of OC patients. Patients known to be still alive at time of analysis were censored at the time of their last follow-up. The status of overall survival regarding ITGA5 expression was estimated with the Kaplan–Meier method using GraphPad Prism 6 software, and the log-rank test was used to assess statistical significance.

### Suspended spheres formation

Briefly, tumor cells and primary CAFs were collected and washed with cold PBS, then seeded in ultra-low attachment plates (Corning Life Sciences) and cultured in complete medium or malignant ascites supernatant (depleted of cellular components through fierce centrifugation) at 37°C for 48 h. To assess development of heterotypic spheroids, GFP-transfected tumor cells were mixed with PKH-26 (Sigma) labeled primary CAFs (ratio 1:2) in malignant ascites supernatant in ultra-low attachment plates. After overnight culture at 37°C, heterospheroid formation was observed and counted under a fluorescence microscope (Olympus) the next day. The number of cells contained in heterotypic spheroids formed in variant conditions was counted next.

### 3D invasion assay

Spheroids were embedded in collagen type I (BD) in 96-well plates. The collagen plugs containing the spheroids were incubated in complete DMEM/F-12 medium for various times. Spheroid invasion was observed by fluorescence microscopy. Invasive surface areas were quantified using ImageJ software.

### Flow cytometry, cell sorting, cell viability, and anoikis assays

For flow cytometry, all antibodies were purchased already conjugated with fluorescent dyes except the anti-mouse FAP antibody. A PercP-conjugated goat anti-rabbit IgG secondary antibody (Novus; NB7156PCP) was used for mouse FAP detection. To compare variant cell population in ascites of HGSOC and LGSOC patients, ascites samples from HGSOC and LGSOC patients were mildly centrifuged to obtain ascites cells. Then, all ascitic tissues were subjected to RBC lysis buffer (BioLegend) followed by brief trypsinization to dissolve aggregates. Cells were first gated based on forward and side scatter to exclude debris. Dead cells were excluded based on their positive staining for DAPI (Thermo Fisher Scientific; D1306). Ascitic live cells were first gated with APC-conjugated EpCAM (BioLegend; 324208) and PC5.5-conjugated CD45 (BioLegend; 368504). EpCAM^−^CD45^−^ cells were then gated with PE-conjugated CD31 (BioLegend; 303106), and EpCAM^−^CD45^−^CD31^−^ cells were finally gated with Alexa Fluor 488–conjugated FAP (R&D Systems; FAB3715G) to identify fibroblast percentage.

In the orthotopic and i.p. implantation model of GFP-transfected SKOV3 cells, peritoneal heterospheroids were obtained, and GFP^−^ host cells were sorted on a BD FACSAria SORP for further identification of variant cell types. The selected GFP^−^ cells were first gated with PE-conjugated EpCAM (BioLegend; 118206) and APC-conjugated CD45 (BioLegend; 103112). EpCAM^−^CD45^−^ cells were then gated with FITC-conjugated CD31 (BioLegend; 102406), and EpCAM^−^CD45^−^CD31^−^ cells were finally gated with PercP-conjugated FAP (Abcam; ab28244) to identify fibroblasts. As for the i.p. implantation model of PKH26-labeled ID8 cells in the EGFP mouse, peritoneal heterospheroids were obtained, and GFP^+^ host cells were sorted for further identification. The selected GFP^+^ cells were first gated with PE-conjugated EpCAM and APC-conjugated CD45/CD31 (BioLegend; 103112/102410), and EpCAM^−^CD45^−^CD31^−^ cells were then gated with PercP-conjugated FAP to identify fibroblasts. Corresponding isotype control antibody was used for each antibody in all experiments. All cell samples were run on a FACS Calibur system (Becton Dickinson) and analyzed using CytExpert 2.0 software.

Cell viability was determined using the Cell Counting Kit-8 (Dojindo Laboratories; CCK-8, CK04). For cytotoxicity assays with imatinib (Selleck), 5,000 adherent tumor cells or CAFs per well were plated in 96-well plates and left to adhere before various concentrations of imatinib were applied for 48 h. The CCK-8 solution (10 µl) was added to each well and incubated for another 3 h at 37°C before absorbance at 450 nm was measured on a microplate reader (Bio-Rad). Cell viability was calculated based on the absorbance value relative to untreated control cells. Each assay was performed in triplicate.

For anoikis analysis, 2 × 10^5^ single GFP^+^ SKOV3 cells were allowed to form homospheroids or heterospheroids with CAFs, in the presence or absence of imatinib (20 nM) before being seeded onto 6-well ultra-low attachment plates. After 24 or 48 h, cells were harvested and incubated at 37°C with 0.25% trypsin for 5 min to prevent cell aggregation. Apoptotic GFP^+^ cells were detected by annexin V-PE (BioLegend; 640908) staining using a FACS Calibur system (Becton Dickinson).

### EGF detection by ELISA

EGF protein levels in patient malignant ascites and CM of CAFs were measured by ELISA kits (R&D Systems) according to the manufacturer’s protocol. For detection of EGF in spheroids, spheroids were disaggregated with 0.25% trypsin (Thermo Fisher Scientific) and vigorous pipetting before the ELISA measurement.

### Western blot and human antibody array

Cells were collected and washed with PBS and then lysed with RIPA lysis buffer (Beyotime) supplemented with a protease inhibitor cocktail (Roche). Total protein amount was measured with a bicinchoninic acid assay (Thermo Fisher Scientific), and 40 µg total lysate per sample was subjected to SDS-PAGE followed by immunodetection with the following primary antibodies: ITGA5 (Abcam; ab150361), ITGB1 (Abcam; ab52971), CDH1 (Abcam; ab40772), Vim (Abcam; ab92547), GAPDH (Abcam; ab128915), CK7 (Abcam; ab181598), PDGFRβ (Abcam; ab32570), α-SMA (Abcam; ab7817), and FAP (Abcam; ab28244). For detection, the corresponding HRP-linked secondary antibody (Abcam) and enhanced chemiluminescence (Pierce) were added. Human antibody arrays were performed according to the manufacturer’s instructions. Briefly, human primary CAFs were pretreated with SKOV3-derived CM or 50 ng/ml TGF-β1 (Peprotech) for 48 h, then cultured in serum-free medium for another 48 h. Culture supernatants were collected and assayed using the human cytokine antibody array AAH-CYT-G5 (RayBiotech). Signals were detected using a GenePix 4200A Professional microarray scanner.

### Immunohistochemistry, Masson’s trichrome staining, and immunofluorescence

OC tissues from primary tumors and metastatic sites in advanced OC patients, as well as normal ovaries and fallopian tubes, were obtained from patients who had undergone surgery at the Department of Gynecological Oncology of Tongji Hospital. Specifically, cancer tissues were from patients diagnosed with advanced (stages III and IV) serous adenocarcinoma based on a pathological evaluation. Normal ovaries and fallopian tubes were obtained from patients who underwent prophylactic adnexectomy due to benign uterine lesions such as multiple adenomyoma. Informed consent was obtained from all patients. Sample sizes were chosen by power analysis. Immunohistochemical staining for ITGA5 in tumor tissues, as well as EGF and EGFR in sequential ovaries, fallopian tubes, primary tumors, and metastases slices were stained as previously described (Greenblatt et al., 2015). Briefly, antigen retrieval was performed in the presence of 0.01 mol/liter EDTA buffer (pH 9.0). After 30 min of blocking in goat serum (Dako), the slides were incubated overnight with primary antibody (ITGA5, ab150361; EGF, ab9695; EGFR, ab52894; Abcam) at 4°C followed by an HRP-linked secondary antibody for 30 min. The slides were then developed using the DAB kit (BD Biosciences) for optimal staining intensity. Three blinded investigators scored the immunostainings based on the staining intensity and the positively stained areas, as previously described (Liu et al., 2014). These data were analyzed as a continuum, and differences between groups were compared with this semiquantitative method.

Masson’s trichrome (Sigma) staining of paraffin-embedded sections of mouse xenografts was performed as previously described (Shen et al., 2014). Dual immunofluorescence for α-SMA (Abcam; ab7817), PDGFRβ (Abcam; ab69506), or prolyl 4-hydroxylase (Thermo Fisher Scientific; MA3-019) with EpCAM (Abcam; ab213500) on HGSOC ascitic spheroids was performed as follows: frozen sections of ascitic tissues were washed with PBS, fixed in 4% paraformaldehyde (Sigma) for 10 min, permeabilized with 0.1% Triton X-100 (Roche), blocked with 5% BSA, stained with a primary antibody for α-SMA/PDGFRβ/prolyl 4-hydroxylase in combination with anti-EpCAM, and then stained with anti-rabbit Alexa Fluor 488 and anti-mouse Alexa Fluor 555 (Thermo Fisher Scientific) for 30 min. The nucleus was visualized by staining with DAPI. Representative images were acquired using a fluorescence microscope (Olympus).

### *ITGA5* promoter reporter assay

The human *ITGA5* promoter region (−908/+241) was generated by high-fidelity PCR with primer set (5′-CCGCTCGAGGAGCTGAAGGTTGGGTCC-3′ and 5′-CCGCTCGAGCCGTCTGTTCCCGGC-3′), using genomic DNA from SKOV3 cells. The PCR product was digested and cloned into the PGL3 basic vector (Promega) to generate PGL3-*ITGA5*-Luc construct. Tumor cells grown in 10-cm culture dishes were transiently cotransfected with 10 µg reporter plasmid and 1 µg *Renilla* luciferase vector (pRL-TK; Promega) for 36 h. Tumor cells were then digested and subjected to spheroid coculture with primary CAFs in variant conditions for 48 h. Subsequently, luciferase activity was evaluated using the dual-luciferase reporter assay system (Promega) in isolated tumor cells, and the data were normalized with *Renilla* luciferase.

### CRISPR/Cas9 editing of *ITGA5* and *EGFR* in cancer cells

Open-access software program CRISPR design was used to design sgRNAs targeting *ITGA5* and *EGFR*. The three highest ranking gRNAs were chosen per gene. Three sgRNA sequences for human *ITGA5* are as follows: sgRNA1: 5′-GGGGCAACAGTTCGAGCCCA-3′, sgRNA2: 5′-GGAGCCACTGAGCGACCCCG-3′, and sgRNA3: 5′-TCTGTGCGCCAGCTGTACA G-3′. Three sgRNA sequences for human *EGFR* are as follows: sgRNA1: 5′-GAATTCGCTCCACTGTGTTG-3′, sgRNA2: 5′-TGTGATCCAAGCTGTCCCAA-3′, and sgRNA3: 5′-GACAGCTTGGATCACACTTT-3′. The scrambled sgRNA sequence is 5′-GCGCCAAACGTGCCCTGACG-3′. sgRNAs oligos were purchased from Vigene Biosciences and then cloned into the Cas9 backbone LentiCRISPR_v2 (Addgene; 52961) according to the manufacturer’s instructions (Ran et al., 2013). Lentivirus production and cancer cell infection were performed as previously described (Taylor et al., 2013). The extent of *ITGA5* or *EGFR* deficiency was first determined by T7E1 assays, as described previously (Kim et al., 2009a). Immunoblotting was then performed in stable pools of *ITGA5*- or *EGFR*-deficient SKOV3 cells obtained by culture over 10 d in 4 µg/ml puromycin (Sigma). The primers used for PCR amplification of the targeting site of *ITGA5* and *EGFR* are as follows: *ITGA5*-sgRNA1: 5′-CGTGTGTATGTATGTGTGTGTGTG-3′ (forward) and 5′-GCTCAGTGGCTCCTTCTCTG-3′ (reverse); *ITGA5*-sgRNA2/3: 5′-CTCAAATGTCCATGGCTCAG-3′ (forward) and 5′-ATGGTAGGTGCGAGTCAACC-3′ (reverse); *EGFR*-sgRNA1: 5′-GTGAGGCATGAGAGCACAGT-3′ (forward) and 5′-CGAGGTGGAATTGAGTGACA-3′ (reverse); and *EGFR*-sgRNA2/3: 5′-CTTGCTTATGTGGCCCATGT-3′ (forward) and 5′-AACTGCATGCGGTGAGATTT-3′ (reverse).

### Animal experiments

Animal studies were performed with the approval of the Committee on the Ethics of Animal Experiments in Hubei Province. 6–8-wk-old female β-actin EGFP transgenic mice (C57BL/6-Tg (CAG-EGFP)10sb/J) were transferred from the Institute of Zoology, Chinese Academy of Sciences. 4–6-wk-old female NOD/SCID and BALB/c nude mice were purchased from HFK Bioscience Co. All mice were bred and maintained in laminar flow cabinets under specific pathogen–free conditions.

To assess the effects of loss of ITGA5 or EGFR on OC progression, control or ITGA5/EGFR-deficient SKOV3-Luc cells (5 × 10^6^) were implanted into the peritoneal cavity of NOD/SCID mice. Tumor growth was monitored weekly since tumor inoculation. To analyze the influence of HGSOC-derived CAFs exertion on LGSOC ATCs metastasis, LGSOC ATC cells (6 × 10^6^) were injected i.p. either alone or with HGSOC-derived CAFs (6 × 10^6^) in NOD/SCID mice. Mice were observed every 2 d, and body weight was evaluated twice weekly throughout the intervention period. To analyze the effect of EGF blocking on OC dissemination, SKOV3-Luc cells (4 × 10^6^) were injected together with primary CAFs (4 × 10^6^) into the peritoneal cavity of NOD/SCID mice. Mice were then randomized to treatment groups receiving 10 mg/kg IgG or EGF neutralizing antibody (R&D Systems) twice weekly, starting from the time of implantation. To analyze the influence of imatinib priming of CAFs on OC dissemination, SKOV3-Luc cells (4 × 10^6^) were injected either alone or together with control or imatinib-primed CAFs (4 × 10^6^) into the peritoneal cavity of NOD/SCID mice. In the parallel orthotopic metastatic model, SKOV3-Luc cells (4 × 10^6^) were injected orthotopically and randomized to different treatment cohorts, treated i.p. with 100 mg/kg imatinib (in PBS) daily during the initial week (early intervention group), or treated from 1 wk until the end of the experiment (late intervention group). To further analyze TAM depletion influence on OC dissemination, BALB/C nude mice orthotopically inoculated with SKOV3-Luc cells (4 × 10^6^) were randomized to different treatment cohorts, treated i.p. with 100 mg/kg imatinib daily during the initial week (ima group) or treated i.p. with 100 µl LC (FormuMax; 40335ES10) every 3 d since tumor inoculation (LC group), or a combination of imatinib and LC as used above (ima+LC group). Control mice received only PBS solution.

All animal experiments were terminated when the mice were restricted in taking in food or drinking water by tumor burdens. Mice were imaged longitudinally with the IVIS Spectrum system (Caliper; Xenogen) 15 min after intraperitoneal administration of 100 mg/kg d-luciferin (Thermo Fisher Scientific). Investigators were blinded for the assessment of the total flux (photons/s) from orthotopic, mesentery, or total peritoneal tumors, which were analyzed using Living Image version 4.3.1 software.

### Statistical analysis

All data including error bars are presented as means ± SEM. All calculations were performed using GraphPad Prism 6.0. Two experimental groups were compared by using a paired Student’s *t* test for paired data or a Student’s *t* test for unpaired data. Where more than two groups were compared, a one-way ANOVA with Bonferroni’s correction was used. P < 0.05 was considered significant.

### Online supplemental material

Fig. S1 depicts ATCs in comparison with matched counterpart tumor cells in LGSOC. Fig. S2 shows the aggressive behavior of CAF-centered spheroids. Fig. S3 depicts fibroblasts contributing to MU formation in various murine models. Fig. S4 shows EGF and EGFR expression pattern in OC tissues. Fig. S5 indicates that early intervention with imatinib or eradication of TAMs prevents peritoneal dissemination in orthotopic mouse models of OC. Tables S1, S2, and S3 show genes significantly dysregulated among ATCs and primary and metastatic tumor cells in HGSOC patients. Table S4 includes genes significantly up-regulated in SKOV3 cells that formed heterotypic spheroids with CAFs compared with those remained individual SKOV3 cells. Table S5 shows cytokine profiles of CM from CAFs in the control group, or from CAFs pretreated with SKOV3-CM or TGF-β1. Table S6 shows a summary of OC patient clinical data.

## Supplementary Material

Supplemental Materials (PDF)

Tables S1-S6 (XLSX)
