# Correction: Heterotypic CAF-tumor spheroids promote early peritoneal metastatis of ovarian cancer

**DOI:** 10.1084/jem.2018076508222019c

**Published:** 2019-08-26

**Authors:** Qinglei Gao, Zongyuan Yang, Sen Xu, Xiaoting Li, Xin Yang, Ping Jin, Yi Liu, Xiaoshui Zhou, Taoran Zhang, Cheng Gong, Xiao Wei, Dan Liu, Chaoyang Sun, Gang Chen, Junbo Hu, Li Meng, Jianfeng Zhou, Kenjiro Sawada, Robert Fruscio, Thomas W. Grunt, Jörg Wischhusen, Víctor Manuel Vargas-Hernández, Bhavana Pothuri, Robert L. Coleman

Vol. 216, No. 3, March 4, 2019. 10.1084/jem.20180765.

The authors regret that in their original paper, the Masson’s image of the control group in Fig. 7 J was incorrect as a result of an error during figure preparation. The corrected figure appears below.

**Figure 7.**
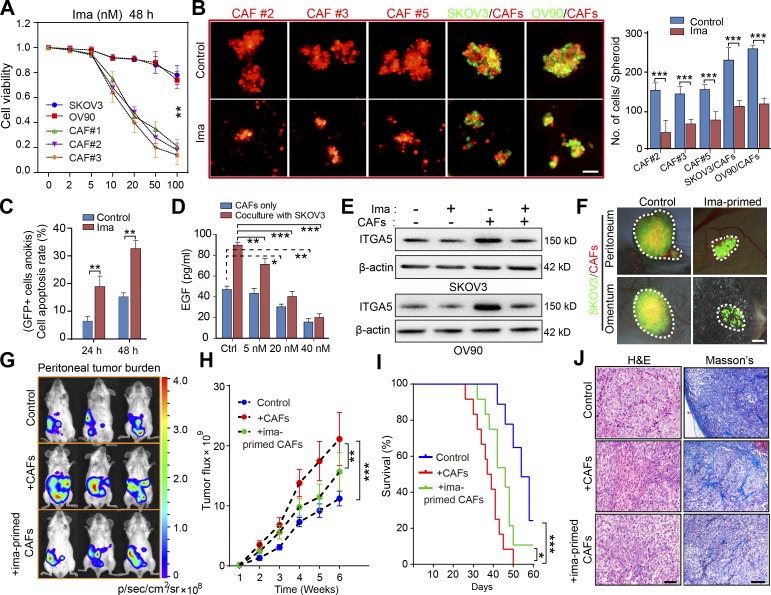
Targeting CAFs in ascites disrupts MUs and attenuates OC dissemination. **(A)** Cell viability analysis of the OC tumor cells and of primary CAFs (#1–3) exposed for 48 h to varying doses of imatinib (ima; 0–100 nM). **(B)** Representative images and quantification of spheroid formation by primary CAFs (#2, 3, and 5) or in suspended coculture with GFP-transfected tumor cells, in the presence or absence of 20 nM imatinib. Bar, 50 µm. **(C)** Flow cytometry analysis of cellular apoptosis rate for GFP^+^ SKOV3 cells cultured in heterotypic spheroids with CAFs (#2, 5, and 6) for the indicated time, in the presence or absence of 20 nM imatinib. **(D)** EGF secretion by CAFs (#3, 5, and 8) cocultured or not with SKOV3 cells, in the presence or absence of varying doses of imatinib, was assessed by ELISA. **(E)** Immunoblot of ITGA5 in control SKOV3 and OV90 cells, or after heterotypic coculture with CAFs (#5 and 8), in the presence or absence of 20 nM imatinib. **(F)** Representative images of peritoneal sphere adhesion 1 wk after coimplantation of SKOV3 cells with ima-primed CAFs (#3, 7, and 8) or untreated controls. Bar, 50 µm. **(G–I)** Representative bioluminescence images (G), tumor growth curves (H), and survival curves (I) in SKOV3-Luc tumor-bearing mice coimplanted with imatinib-primed CAFs (#7 and 8) or untreated controls (*n* = 10 mice per group). **(J)** H&E and Masson’s trichrome staining of tumors from mice implanted with SKOV3-Luc cells only or coimplanted with ima-primed or untreated CAFs. Bars, 50 µm (left) and 100 µm (right). Data are means ± SEM and representative of four (A–C), two (D, E, and G–J) or three (F) independent experiments. *, P < 0.05; **, P < 0.01; ***, P < 0.001; determined by Student’s *t* test.

The online HTML and PDF have been corrected. The error remains only in the print version.

