# Supplementary material for: Heterotypic CAF-tumor spheroids promote early peritoneal metastatis of ovarian cancer
Source: J Exp Med. 2019 Mar 4;216(3):688–703. doi: 10.1084/jem.20180765 (PMC6400537; doi:10.1084/jem.20180765)
Supplement: Supplemental Materials (PDF) [file JEM_20180765_sm.pdf]

Supplemental material

Gao et al., https://doi.org/10.1084/jem.20180765

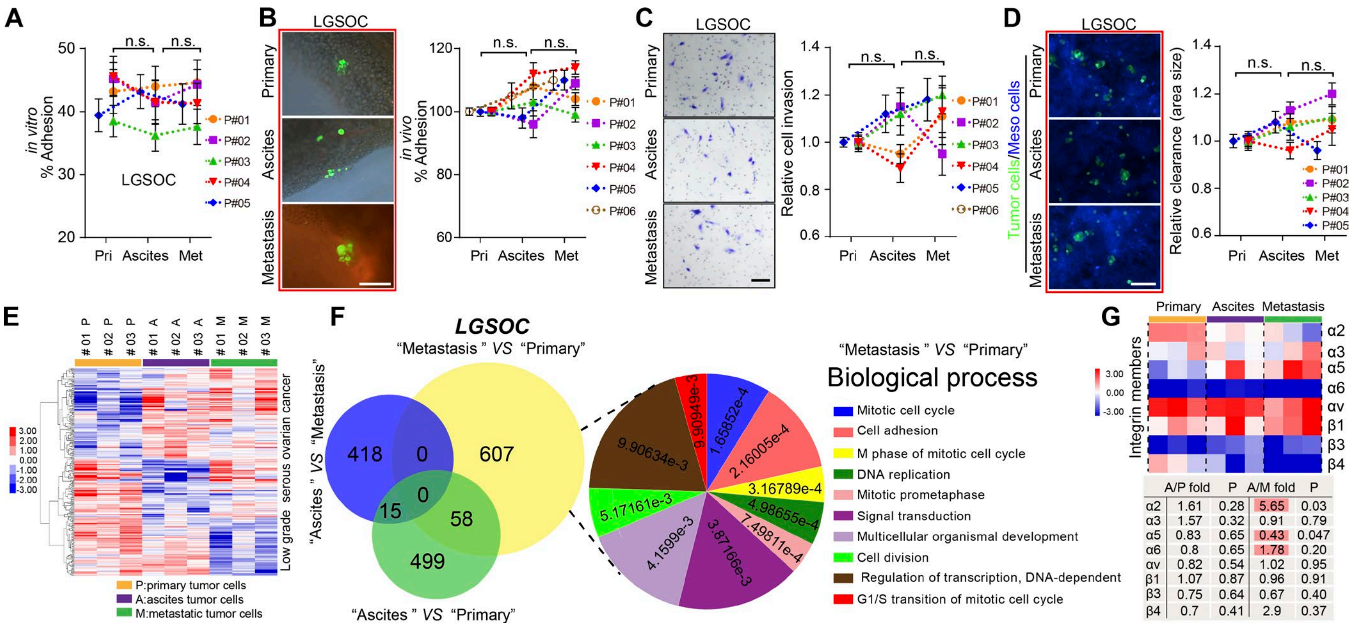

Figure S1. **ATCs in comparison with matched counterpart tumor cells in LGSOC. (A)** Relative percentage of paired tumor cells from primary tumors (Pri), ascites, and metastases (Met) adhesion to the major ECM protein fibronectin in LGSOC patients (#01–05). **(B–D)** Representative images and quantification of paired tumor cell adhesion in murine abdominal cavity (B), tumor cell invasion (C) in LGSOC patients (#01–06), and mesothelial clearance in LGSOC patients (#01–05; D). Bar, 50 µm. **(E)** Hierarchical clustering of differentially expressed genes among nine epithelial tumor cell specimens purified from primary tumors (P), ascites (A), and metastases (M) of three LGSOC patients. **(F)** Venn diagram of the common signature genes differentially expressed between tumor cells of different origins and major biological processes represented in the gene sets specifically elevated in the metastatic tumor cells from LGSOC with their corresponding statistical significances. **(G)** Expression levels of relevant integrin family members were extracted from the microarray data and compared according to tumor cell origin in LGSOC. Data are means ± SEM. Data are one experiment (E–G) and representative of four (A–D) independent experiments. n.s., no significance; determined by paired Student's *t* test.

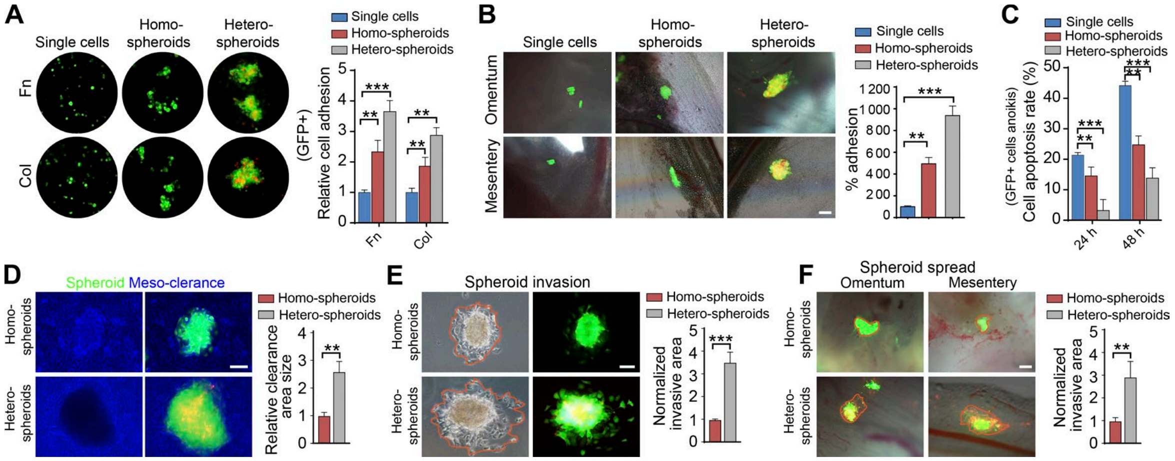

Figure S2. **CAF-centered spheroids exhibit higher malignant potential. (A and B)** Representative images and quantification of in vitro (A) and in vivo (B) adhesion for GFP-transfected SKOV3 cells in the form of single cells, homospheroids, and CAF-centered heterospheroids. Results were presented as numbers of adhering cells relative to the single-cell group. Bar, 50 µm. **(C)** Flow cytometry analysis of apoptosis in GFP^+ SKOV3 cells as single cells, homospheroids, and CAF-centered heterospheroids after culture in suspension for the indicated times. **(D)** Representative images of homospheroids and heterospheroids subjected to mesothelial clearance assays. Result was shown as clearance area size compared with that in the homospheroid group. Bar, 50 µm. **(E and F)** Representative images and quantification of homo- and heterospheroid invasion in 3D gel assays (E) and in vivo spreading analysis (F). Bar, 50 µm. Results are presented as normalized invasive area size compared with the homospheroid group. Data are means ± SEM and are representative of four (A–C) or three (D–F) independent experiments. **, $P < 0.01$; ***, $P < 0.001$, determined by Student's *t* test.

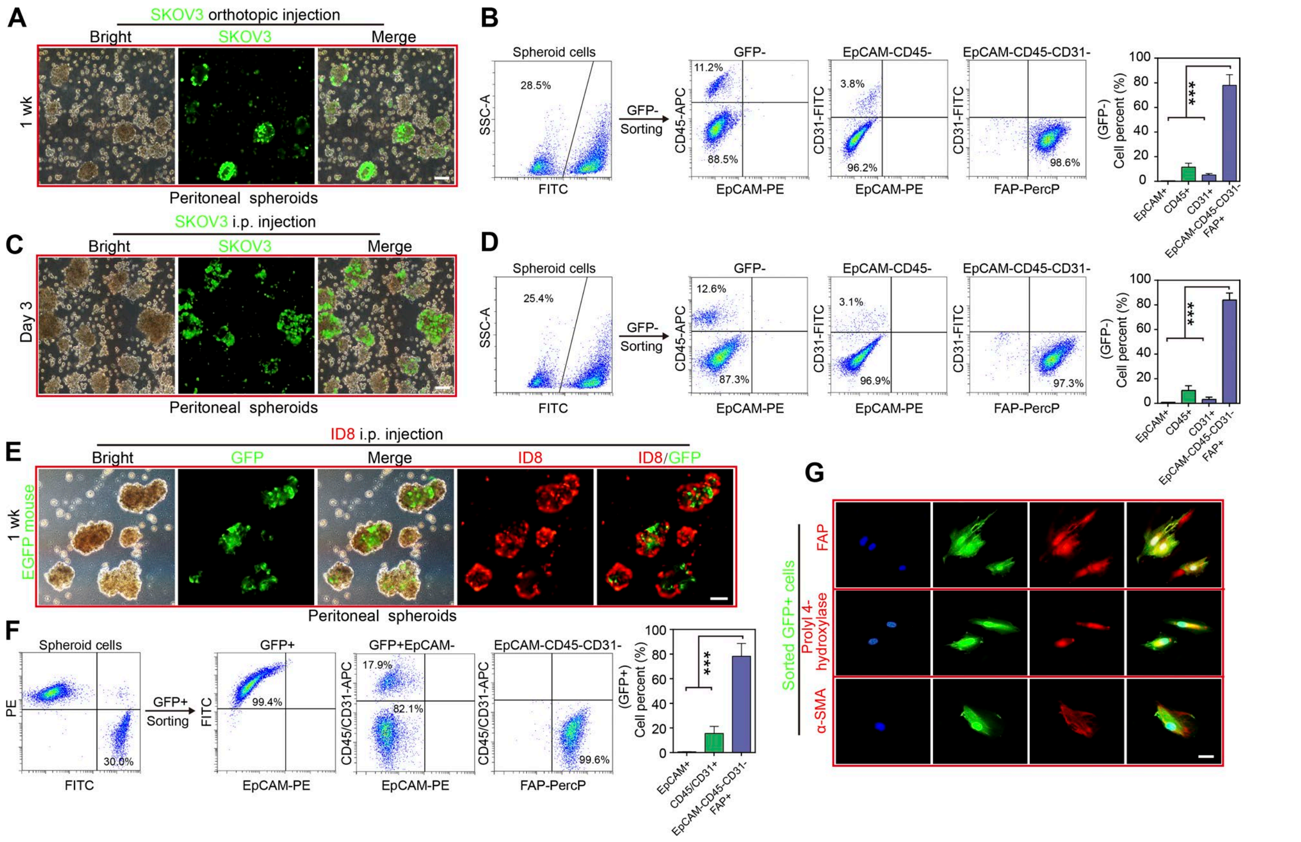

Figure S3. **Host fibroblasts contribute to MU formation in various murine models. (A)** Representative images of abdominal heterotypic spheroids collected 1 wk after orthotopic implantation of GFP-transfected SKOV3 cells. Bar, 100 µm. **(B)** Representative flow cytometry plots (left) and frequencies (right) showing expression percentage of EpCAM$^+$ tumor cells, CD45$^+$ leucocytes, CD31$^+$ endothelial cells, and EpCAM$^-$CD45$^-$CD31$^-$FAP$^+$ fibroblasts among those isolated GFP$^-$ subpopulations from heterotypic spheroids developed in A. **(C)** Representative images of abdominal heterotypic spheroids collected on day 3 after i.p. injection of GFP-transfected SKOV3 cells. Bar, 100 µm. **(D)** Representative flow cytometry plots (left) and frequencies (right) showing expression percentage of EpCAM$^+$ tumor cells, CD45$^+$ leucocytes, CD31$^+$ endothelial cells, and EpCAM$^-$CD45$^-$CD31$^-$FAP$^+$ fibroblasts among those isolated GFP$^-$ subpopulations from heterotypic spheroids developed in C. **(E)** Representative immunofluorescence images of peritoneal heterotypic spheroids 1 wk after i.p. implantation of PKH-26–labeled ID8 tumor cells in β-actin-EGFP mouse. Bar, 100 µm. **(F)** Representative flow cytometry plots (left) and frequencies (right) showing expression percentage of EpCAM$^+$ tumor cells, CD45$^+$/CD31$^+$ leucocytes/endothelial cells, and EpCAM$^-$CD45$^-$CD31$^-$FAP$^+$ fibroblasts among those isolated GFP$^+$ subpopulations from heterotypic spheroids developed in E. **(G)** Immunofluorescence images demonstrating the expression of FAP, prolyl 4-hydroxylase, or α-SMA in the sorted GFP$^+$ host cells included in the heterotypic spheroids in E. Nuclei were counterstained with DAPI. Bar, 20 µm. Data are means ± SEM and are representative of four (A–F) or two (G) independent experiments. ***, $P < 0.001$, determined by one-way ANOVA followed by Bonferroni's post hoc test.

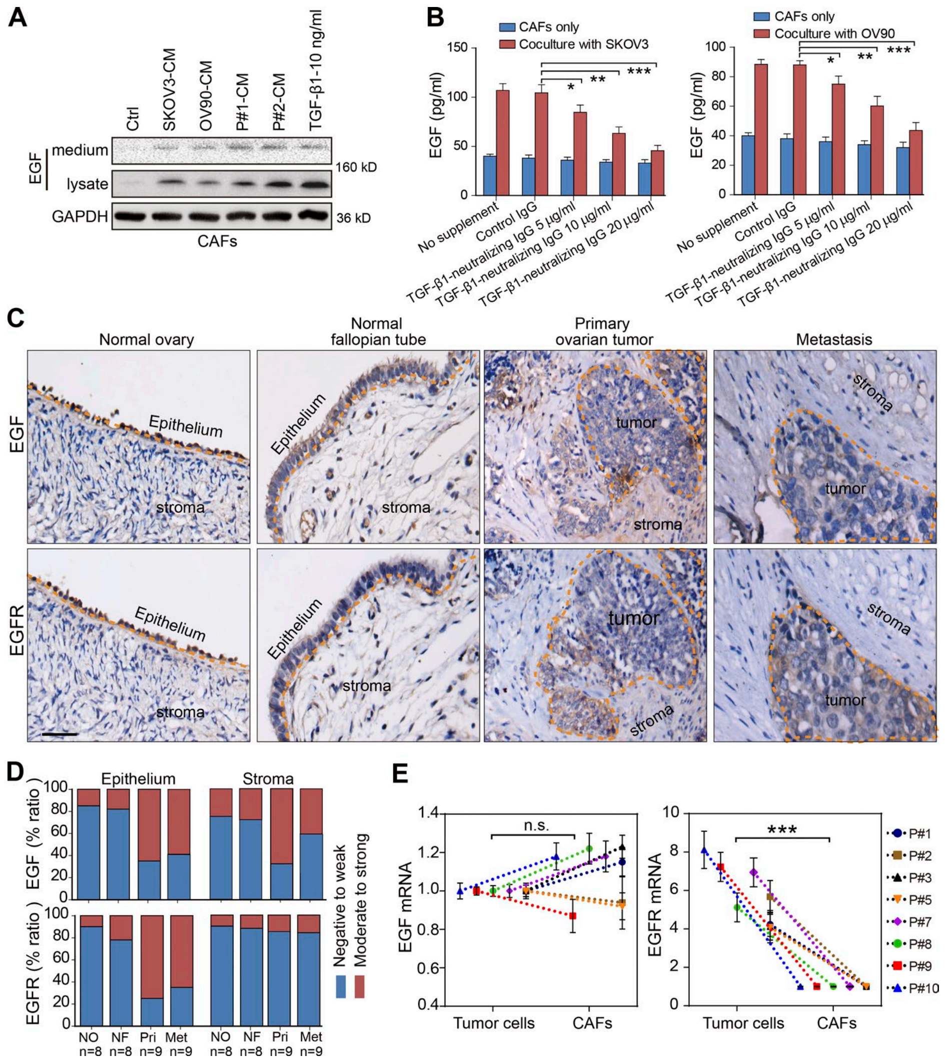

Figure S4. **EGF and EGFR expression pattern in OC tissues. (A)** Immunoblotting for EGF in culture medium from CAFs and in cell lysates, in the absence or presence of tumor cells (SKOV3, OV90, P#1 and 2) CM or TGF-β1. **(B)** EGF secreted from CAFs (#8–10) was analyzed by ELISA when CAFs were solitary or in coculture with tumor cells, in the presence or absence of IgG or increasing amounts of neutralizing anti–TGF-β1 antibody. **(C)** Representative immunohistochemistry images of EGF and EGFR in serial sections of normal ovaries, normal fallopian tubes, primary tumors, and metastases from HGSOC. Bar, 50 µm. **(D)** Scoring of EGF and EGFR expression in the epithelial and stromal components of the tissues stained in C. **(E)** Quantitative PCR analysis of EGF and EGFR in eight pairs of tumor epithelial cells and stromal CAFs purified from fresh HGSOC patient cells. Data are means ± SEM and are representative of two (A–E) independent experiments. *, P < 0.05; **, P < 0.01; ***, P < 0.001; n.s., no significance; determined by Student's *t* test.

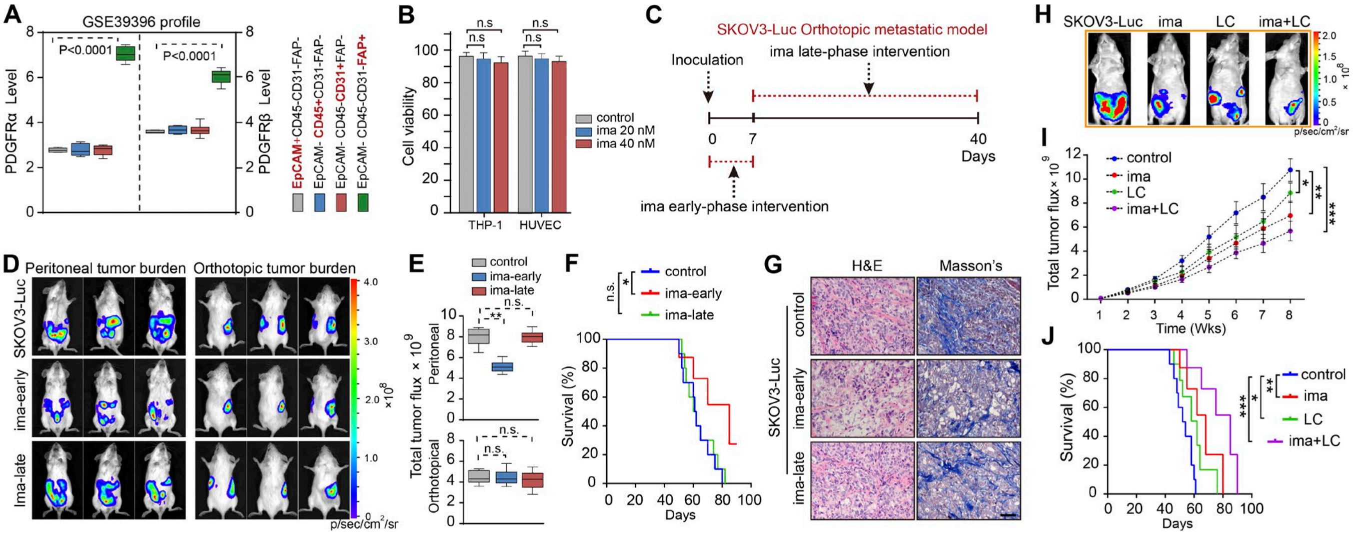

Figure S5. **Early intervention with imatinib (ima) or eradication of TAMs hampers peritoneal dissemination in orthotopic mouse models for OC. (A)** Relative expression of *PDGFRα* and *PDGFRβ* in tumor cells (EpCAM$^+$CD45$^-$CD31$^-$FAP$^-$), leucocytes (CD45$^+$CD31$^-$EpCAM$^-$FAP$^-$), endothelial cells (CD31$^+$EpCAM$^-$CD45$^-$FAP$^-$), and fibroblasts (FAP$^+$EpCAM$^-$CD45$^-$CD31$^-$) resident in the tumor microenvironment, as extracted from the GSE39396 profile. Statistical significance was determined by one-way ANOVA. **(B)** Cell viability analysis of THP-1 and HUVEC cells exposed to 20 nM and 40 nM imatinib for 48 h. **(C)** Scheme depicting the administration of imatinib in the SKOV3-Luc orthotopic xenograft model in NOD/SCID mice. **(D–F)** Representative bioluminescence images (D), quantification of the peritoneal and orthotopic tumor burden (E), and survival curves (F) in SKOV3-Luc tumor-bearing mice in control and in imatinib-treated (early and late) groups (n = 10 mice per group). **(G)** H&E and Masson's trichrome staining of tumors from mice in the control, imatinib-early- and imatinib-late-intervention groups. Bar, 50 µm. **(H–J)** Representative bioluminescence images (H), growth curves (I), and survival curves (J) in BALB/c nude mice orthotopically inoculated with SKOV3-Luc cells in control, imatinib, LC, and imatinib in combination with LC (ima+LC) groups (n = 10 mice per group). Data are means ± SEM. Data are one experiment (A) and representative of three (B) or two (D–J) independent experiments. *, $P < 0.05$; **, $P < 0.01$; ***, $P < 0.001$; n.s., no significance; determined by Student's t test.

Tables S1–S6 are provided as a single Excel file. Tables S1, S2, and S3 show genes significantly dysregulated among ATCs and primary and metastatic tumor cells in HGSOC patients. Table S4 includes genes significantly up-regulated in SKOV3 cells that formed heterotypic spheroids with CAFs compared with those remained individual SKOV3 cells. Table S5 shows cytokine profiles of CM from CAFs in the control group or from CAFs pretreated with SKOV3-CM or TGF-β1. Table S6 shows a summary of OC patient clinical data.
